# The Thing Metabolome Repository family (XMRs): comparable untargeted metabolome databases for analyzing sample-specific unknown metabolites

**DOI:** 10.1093/nar/gkac1058

**Published:** 2022-11-24

**Authors:** Nozomu Sakurai, Shinichi Yamazaki, Kunihiro Suda, Ai Hosoki, Nayumi Akimoto, Haruya Takahashi, Daisuke Shibata, Yuichi Aoki

**Affiliations:** Bioinformation and DDBJ Center, National Institute of Genetics, 1111 Yata, Mishima, Shizuoka 411-8540, Japan; Kazusa DNA Research Institute, 2-6-7 Kazusa-kamatari, Kisarazu, Chiba 292-0818, Japan; Sakura Scientific Co. Ltd., 35 Horinouchi, Odawara, Kanagawa 250-0853, Japan; Tohoku Medical Megabank Organization, Tohoku University, 2-1 Seiryo-machi, Aoba-ku, Sendai, Miyagi 980-8573, Japan; Kazusa DNA Research Institute, 2-6-7 Kazusa-kamatari, Kisarazu, Chiba 292-0818, Japan; Bioinformation and DDBJ Center, National Institute of Genetics, 1111 Yata, Mishima, Shizuoka 411-8540, Japan; Kazusa DNA Research Institute, 2-6-7 Kazusa-kamatari, Kisarazu, Chiba 292-0818, Japan; Division of Food Science and Biotechnology, Graduate School of Agriculture, Kyoto University, Gokasho, Uji, Kyoto 611-0011, Japan; Kazusa DNA Research Institute, 2-6-7 Kazusa-kamatari, Kisarazu, Chiba 292-0818, Japan; Tohoku Medical Megabank Organization, Tohoku University, 2-1 Seiryo-machi, Aoba-ku, Sendai, Miyagi 980-8573, Japan; Graduate School of Information Sciences, Tohoku University, 6-3-09 Aramaki-Aza-Aoba, Aoba-ku, Sendai, Miyagi 980-8679, Japan

## Abstract

The identification of unknown chemicals has emerged as a significant issue in untargeted metabolome analysis owing to the limited availability of purified standards for identification; this is a major bottleneck for the accumulation of reusable metabolome data in systems biology. Public resources for discovering and prioritizing the unknowns that should be subject to practical identification, as well as further detailed study of spending costs and the risks of misprediction, are lacking. As such a resource, we released databases, Food-, Plant- and Thing-Metabolome Repository (http://metabolites.in/foods, http://metabolites.in/plants, and http://metabolites.in/things, referred to as XMRs) in which the sample-specific localization of unknowns detected by liquid chromatography–mass spectrometry in a wide variety of samples can be examined, helping to discover and prioritize the unknowns. A set of application programming interfaces for the XMRs facilitates the use of metabolome data for large-scale analysis and data mining. Several applications of XMRs, including integrated metabolome and genome analyses, are presented. Expanding the concept of XMRs will accelerate the identification of unknowns and increase the discovery of new knowledge.

## INTRODUCTION

A major bottleneck in systems biology is the poor availability of complete datasets of chemical compounds (metabolome) present in the samples. Using high-sensitivity and high-throughput mass spectrometry (MS) in an untargeted manner, several thousands of signals derived from the chemicals are detected simultaneously from a sample. However, it is not possible to identify most of the chemicals because of the limited availability of purified standard chemicals required for the identification. To overcome this issue, bioinformatics methods for predicting chemical structures using the mass spectra of fragmented unknown molecules have been studied eagerly and a prediction accuracy of over 70% has been observed in recent years (1,2). Furthermore, an approach for structural elucidation based on the similarity of mass spectral features to those of known compounds (molecular networking) has been provided by the GNPS consortium (3). However, even when these prediction results are available, there is still a lack of information to help prioritize unknowns from the many candidates for further detailed investigation and identification. To identify a genuinely unknown chemical, the researcher will encounter high costs, for example for purification or organic synthesis and the direct determination of its chemical structure, along with considerable risk for failed identification owing to misprediction. A lack of resources for discovering and prioritizing unknowns for further detailed investigations after their selection by statistical analysis has emerged as a long-standing practical issue in untargeted metabolome studies and is considered a bottleneck in systems biology.

A potential way to discover and prioritize unknowns is to establish a public resource that provides sample-specific localization of the unknowns. Sample metadata such as tissue specificity and taxonomic relationships significantly improves metabolite identification (4). A successful example of the identification of unknowns based on sample specificity was biomarker discovery for specific cancer cells using a database (BinBase) of gas chromatography (GC)–MS-based metabolome data obtained from various samples (5). GC–MS, which is suitable for detecting volatiles and primary metabolites, is advantageous for data comparison by the reproducibility of the fragmentation based on electron ionization and the normalized retention time (retention index). In liquid chromatography (LC)–MS-based metabolomics, which is suitable for detecting a wide variety of liquid-soluble unknowns such as plant-derived secondary metabolites, sample specificity can also be used to discover unknowns. For example, Olivon *et al.* (6) discovered seven bioactive natural compounds by combining information about taxonomy, bioactivity, and MS/MS spectral similarity with in-house LC–MS data from 292 plant species in Euphorbiaceae. We also previously annotated tomato metabolites by comparing untargeted LC–MS data of tomatoes with those from Arabidopsis, *Medicago truncatula*, and *Jatropha curcas* (7). However, a public resource for LC–MS-based untargeted metabolome data has not been established.

A major reason for the lack of public resources is the difficulty of comparing LC–MS data between different studies in the public repositories. A considerable amount of metabolome data obtained from various samples has been accumulated in public repositories; examples include MetaboLights (8) and Metabolomics Workbench (9). However, to the best of our knowledge, there is no report of data mining of unknowns involving extensive use of the registered data across a wide range of samples. A major reason for this is the difficulty of data comparisons between different studies. It is even difficult to judge whether two given data are comparable by checking the analytical methods (metadata) and actual accuracy/resolution of the detector when the data are measured. Searches based on mass spectra and precursor ion mass, such as those provided by MASST (10), foodMASST (11), ReDU (12) and Metabolomics Workbench (9), are powerful tools for finding samples that may contain the queried metabolite. However, the absence and thus sample-specific localization of the queried metabolite cannot be examined using their datasets consisting of mixed conditions. In the case of mass spectra, controlling the dependence on the instruments and conditions, mass spectral quality, and coverage of the metabolite peaks are still aspects to be resolved for comparison. One approach to tackle this is to develop datasets obtained by a uniform condition. The dataset of ∼3600 foods from the Global FoodOmics Project used in foodMASST would be a good example of mass spectral data (13). However, comparable datasets for precursor ion mass data, which would be more advantageous than mass spectra in terms of covering the chemical space, are scarce (7).

Here, we report a series of LC–MS-based untargeted metabolome databases as public resources for discovering and prioritizing unknown metabolites based on their sample-specific localizations as evaluated by the precursor ion mass, retention time, and MS^*n*^ or MS/MS spectra. First, we released the Food Metabolome Repository (FoodMR, http://metabolites.in/foods) in 2017, which contained data from 136 food samples (14). Since then, we have expanded the sample variety for FoodMR up to 222 foods and implemented several new functions, such as search functions based on the mass spectral similarity and neutral loss. Furthermore, we developed another database for plants (Plant Metabolome Repository, PlantMR, http://metabolites.in/plants) and have been developing a database for anything (Thing Metabolome Repository, ThingMR, http://metabolites.in/things) (hereafter, we refer to the three databases as XMRs). We used specific LC–MS instruments and conditions to ensure the data comparison between arbitrary samples. Although the detected compounds are limited by the particular methods applied, we can evaluate the sample-specific localization of an unknown by the mass value of the precursor ion, retention time, and mass spectra of product ions. It is notable that the search, download, and browsing functions of the XMRs are available as application programming interfaces (APIs) to use the data from the other computational programs. The untargeted metabolome data have now been used directly by bioinformatics tools in systems biology. In this report, we briefly introduce the statistics and principal functions of XMRs, presenting examples of their practical use and precautions, and discuss the consequences of expanding the concept of XMRs.

## MATERIALS AND METHODS

### Untargeted metabolome analysis

The details of the data acquisition and processing of untargeted metabolome data deposited to XMRs are provided in the Supplementary Methods. Briefly, a uniform metabolite extraction method and the two LC–MS platforms for FoodMR/PlantMR and ThingMR, respectively (Table 1), were used for data acquisition. PowerGetBatch software (https://www.kazusa.or.jp/komics/software/PowerGetBatch) (14,15) was used for peak detection, characterization, and alignment. The parameter setting files of PowerGetBatch are available on the download page of XMRs.

**Table 1.** General properties of XMRs

Database	FoodMR	PlantMR	ThingMR
Samples targeted	foods	plants	anything
# samples	222	28	734 (July 2022)
LC	Agilent 1100 (Agilent)		Nexera2 (Shimadzu)
Column	C_18_ (Tosoh)		C_18_ (GL Science)
Total elution time	107 min		42 min
Retention time drift *	± ∼1% (1 min)	± ∼2% (2 min)	± ∼1% (0.5 min)
MS	LTQ-FT (Thermo Fisher Scientific)		Compact (Bruker)
MS/MS conditions	MS^2^, MS^3^ by Iontrap MS		MS/MS by Q-ToF
Mass accuracy (precursor ion analysis) *	± 5 ppm		±20 ppm
Mass accuracy (product ion analysis) *	± 0.5 Da		±20 ppm

*Recommended given tolerances for peak search.

### Construction of the database system

The XMR system was developed using SpringBoot (Povital Software, Inc.) and MariaDB 5.5, and run on a Linux server (RedHat EL 7.1). The details of the sample metadata were registered in Metabolonote (http://metabolonote.kazusa.or.jp/) (16) under the accession IDs as follows: FoodMR, SE112-123, SE169-172; PlantMR, SE61, SE198-205; and ThingMR, SE221-226 (July 2022). The peak tables and raw and mzXML-converted mass chromatogram data are available on the download page of the XMRs.

### Statistics for ThingMR

**Figure 1.**
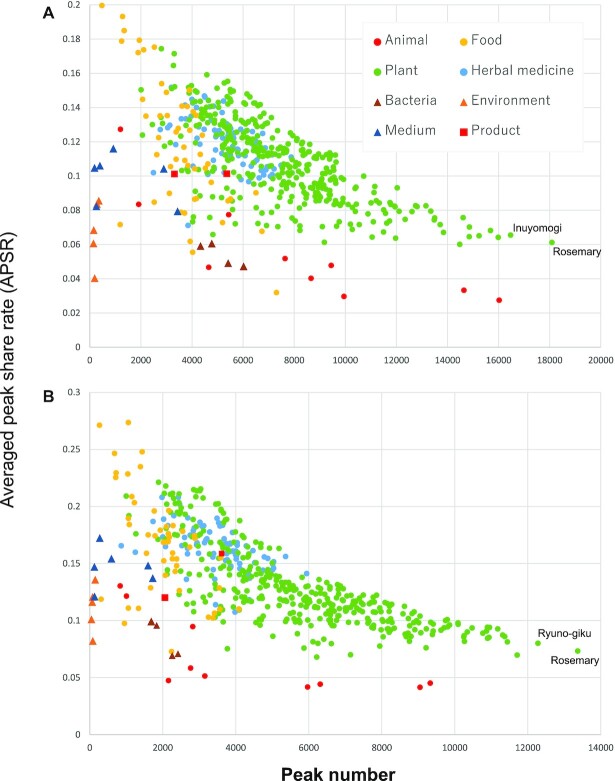
The distribution of averaged peak share rate (APSR) and number of peaks of the samples in FoodMR. The APSR is an index of the richness of sample-specific peaks in the sample. A sample with a lower APSR contains a higher number of sample-specific peaks. (**A**) ESI-positive mode, and (**B**) ESI-negative mode. The values were calculated using 524 samples published in ThingMR (March 2022), except for the standard chemicals.

The peak table consists of the valid peaks detected in 535 samples in ThingMR (March 2022) was constructed using the alignment function of the PowerGetBatch software (14,15) run on the NIG supercomputer, and the results from 524 samples, excluding standard compounds, were used. Each row of the peak table, namely, a set of aligned peaks with the same or similar precursor *m/z* value and retention time in the sample(s), was referred to as a tentative unique peak (TUP). The TUPs per number of samples (Supplementary Figures S1 and S2) were calculated for every 25 samples that were randomly selected from the 524 samples. Ten replicates of the random selection were performed. The peak share rate (PSR) of the TUPs and the average PSR (APSR) of the samples (Figure 1) were calculated as follows:

(1) $$\begin{equation*}{\rm{PSR}} = \frac{{Number\ of\ the\ samples\ where\ the\ TUP\ was\ detected}}{{Total\ number\ of\ the\ samples}}\end{equation*}$$

(2) $$\begin{equation*}{\rm{APSR}} = \frac{1}{{Number\ of\ TUPs}}\sum _{i = 1}^{Number\ of\ TUPs}PS{R}_i\end{equation*}$$

The peak table (alignment results) and parameter setting files of PowerGetBatch were available from the download page of ThingMR. The other statistics were calculated and visualized using Microsoft Excel software (Microsoft Japan Co., Ltd) with the support of in-house Java programs.

### Integrated metabolome and genome analysis

The orthologous gene groups of 80 plant species with scaffold or chromosome-level genome assembly information obtained from NCBI or Ensembl (Supplemental Table S3) were constructed using OrthoFinder version 2.3.12 with default parameters (17). Then, the gene presence/absence profile (where species that retained a gene of interest in their genome were coded as 1, and species that did not have a gene of interest were coded as 0) was calculated for each orthologous gene group. Likewise, the metabolite presence/absence profile (species with a TUP of interest were coded as 1, and species without a TUP of interest were coded as 0) was calculated for 120 plant metabolome data (ESI-positive mode, Supplementary Table S4) in the aligned 535 samples. Then, in order to discover the common phylogenetic distribution patterns between genes and metabolites, an all-to-all similarity comparison of the gene presence/absence profiles and the metabolite presence/absence profiles was performed using in-house Python and R scripts.

### Other methods

The detailed method for datamining for novel flavonoid candidates, statistics for MetagoLights, and identification/annotation procedures for metabolites described in the ‘Application of XMRs’ section are provided in the Supplementary Methods.

## RESULTS

### Development of XMRs

We first established the Food Metabolome Repository (FoodMR, http://metabolites.in/foods) in 2017, with data from food items analyzed in an untargeted manner using reverse-phase LC coupled with high-resolution MS (Table 1) (14). Foods were selected as the target samples because they contain a large variety of chemicals derived from various biological sources and processing techniques, such as mixing, cooking, and fermentation; therefore, the database could be helpful in research fields beyond food science. Since the initial report containing data from 136 foods (14), we have expanded the number of samples to 222. The details of the samples and analytical methods (metadata) are hosted by Metabolonote (16) and therefore searchable through MetabolomeXchange (http://www.metabolomexchange.org/).

We used a uniform analytical method and a data analysis procedure for all samples to ensure arbitrary data comparison and then depict sample-specific localization of the unknowns. The identity or similarity of the peaks was examined by the mass value and retention time at 5 ppm and 1% (∼1 min) tolerances, respectively. In addition, the similarity of the peaks can be examined by their multi-stage mass spectra (MS^2^ and MS^3^) obtained by data-dependent acquisition (DDA) using ion-trap MS if available.

The FoodMR provides some additional information for peak annotation. The compound database search results after searching by the measured mass values and assigned adduct ions of the peaks were available. The following compound databases were used: KEGG (18), KNApSAcK (19), HMDB (20), LIPID MAPS (21) and a flavonoid database (http://metabolomics.jp/wiki/Category:FL). In addition, possible flavonoid aglycones estimated by the FlavonoidSearch tool (https://www.kazusa.or.jp/komics/software/FlavonoidSearch) (22) based on the MS^2^ and MS^3^ spectra were available. For further detailed annotation of the peak, mass chromatogram data converted to mzXML format are available. Using the software supporting the mzXML format, such as MassChroViewer (https://www.kazusa.or.jp/komics/software/MassChroViewer) (14), users can analyze the data and annotate the unknown peaks in detail.

We developed the PlantMR (http://metabolites.in/plants) in 2020 for plant samples, including the inedible parts of plants uncategorized in foods (Table 1). FoodMR and PlantMR are compatible as essentially the same procedures are adopted, although the control of the retention time in PlantMR is not as strict as in FoodMR. Data from 28 plant-related samples, including model plants, Arabidopsis, rice, *Lotus japonicas*, tomato, cultured tobacco cells, *Physcomitrella patents*, *Marchantia polymorpha*, and poplar are available. Some samples contain the predicted atom numbers of nitrogen and sulfur in the chemical structure of the peaks estimated by the comparison with fully labeled plant samples using ^15^N or ^34^S. This information helps the metabolite annotation.

Since 2021, we have been developing the ThingMR (http://metabolites.in/things), suitable for any samples, because—as mentioned later—the compound annotation is improved synergistically by enlargement of sample variety. Four bio-resource centers that joined the National Bio-Resource Project (NBRP, https://nbrp.jp/en/resource-search-en/) in Japan provided 132 samples from basic strains of model organisms (July 2022). We used another LC–MS condition for constructing ThingMR because we could not add new data to FoodMR and PlantMR owing to discarding the LC–MS instrument used. Therefore, the data in ThingMR are not fully compatible with those in FoodMR and PlantMR (Table 1). Although the MS^3^ spectra are not available in ThingMR, the mass accuracy of the MS/MS fragmentation is higher (<20 ppm by Q-ToF MS) than in FoodMR and PlantMR (<0.5 Da by ion-trap MS). This is advantageous for compound prediction based on the mass spectra. Although the column, solvent, and gradient program applied for ThingMR differed from those applied for FoodMR and PlantMR, we can approximate the retention time of the peak based on the regression curve of the retention time of peaks commonly detected in similar samples. Therefore, we can search the peaks by specific *m/z* values and retention times across the three databases (see the section ‘Peak search by mass value and retention time’ and Figure 3).

### Statistics

The statistics for the peaks characterized in the XMRs are summarized in Table 2. More than 4200 peaks were detected on average in a single sample in both ESI-positive and ESI-negative modes. Among them, 14%–21% and 7% had fragmentation mass spectral information in Food/PlantMR and ThingMR, respectively. The higher rate in Food/PlantMR might be attributed to, in part, a longer gradient time and the analytical replicates. The peak ratios assigned as [M + H]^+^ or [M-H]^−^ were lower in Food/PlantMR (78–83%) than in ThingMR (91%–97%). The ratios of the peaks with compound database search results were also lower in Food/PlantMR (36–46%) than in ThingMR (79%–84%). These differences are attributed to the higher mass resolution in Food/PlantMR and the consequently lower mass tolerances given for the search (Table 1). A higher rate of the peaks with FlavonoidSearch results was observed for MS^3^ spectra than for MS^2^ spectra in both FoodMR and PlantMR. This result is in agreement with the concept that modifications such as glycosylation contributed to the increased diversity of flavonoids (23).

**Table 2.** Statistics of the number of peaks in XMRs

Database	FoodMR				PlantMR				ThingMR			
# sample	222				28				734 (July 2022)			
	Positive		Negative		Positive		Negative		Positive		Negative	
# peaks	9 69 352		9 44 312		1 39 953		1 53 769		52 43 731		36 56 502	
# peaks per sample (average)	4366		4254		4998		5492		7144		4982	
# peaks with MS2 or MS/MS spectrum	1 57 697	(16%)	1 28 018	(14%)	29 917	(21%)	23 767	(15%)	3 52 242	(7%)	2 39 035	(7%)
# peaks with MS3 spectra	83 739	(9%)	-		14 024	(10%)	9 138	(6%)	-		-	
# peaks predicted as [M + H]^+^ or [M-H]^−^	7 59 165	(78%)	7 80 548	(83%)	1 11 254	(79%)	1 26 888	(83%)	47 65 869	(91%)	35 33 782	(97%)
# peaks with database search results	3 48 638	(36%)	3 68 545	(39%)	63 866	(46%)	64 860	(42%)	41 67 460	(79%)	30 60 639	(84%)
# peaks with FlavonoidSearch results*	36 646	(4%)	-		7 436	(5%)	-		56 199	(1%)	-	
for MS^2^**	19 854	(13%)	-		4 249	(14%)	-		56 199	(16%)	-	
for MS^3^**	23 396	(28%)	-		4 338	(31%)	-		-		-	

*Parentheses show the ratio to the number of peaks.

**Parentheses show the ratio to the number of peaks with MS^2^ or MS^3^ spectra.

#### An unsaturated number of peaks

We examined the increase in the number of peaks per the increase in the number of samples. Using the data from 535 samples in ThingMR (March 2022), we aligned the same or similar peaks between the samples and created a peak table. In total, 667 417 and 461 121 alignments (referred to as TUPs) assigned as [M + H]^+^ for ESI-positive and [M-H]^−^ for ESI-negative modes, respectively, were generated from the peak data of 524 samples except those of standard chemicals. Using the data, the number of TUPs was counted in randomly selected samples (Supplementary Figure S1). The increase in TUPs was rapid from 2 to ∼200 samples, and then occurred gradually till 400 samples, and finally became almost linear at rest. This result suggested that the variety of detectable chemicals is not saturated with 524 samples. The expected increase in unique peaks per addition of a new sample was estimated in two ways. The first is based on the regression lines calculated with the data obtained from 400–524 samples (Supplementary Figure S1). The slopes of the regression lines suggested that approximately 666 and 450 unique peaks were included in a single sample in positive and negative modes, respectively. The second is based on the distribution of TUPs commonly detected in the samples. As shown in Supplementary Figure S2, the distribution strongly followed the power laws. Therefore, using the number of TUPs detected in only a single sample (326,396 for ESI-positive and 221,996 for ESI-negative modes), we calculated the average TUPs per sample as 623 and 424 for ESI-positive and ESI-negative modes, respectively. These results were in good agreement with the first estimation.

#### The uniqueness of the chemical profile of the sample

To represent the uniqueness of the chemical profile, we defined averaged peak share rate (APSR) of the samples. The PSR, defined as the rate of samples where a TUP was detected to the total sample number (524) was calculated for each TUP. Then, the averaged value of the PSRs of the TUPs detected in a sample was defined as APSR. Therefore, the sample with a smaller APSR would have a larger number of sample-specific peaks (Supplementary Table S1, Figure 1). The samples with smaller APSRs included animal samples (ragworms, urine of cat and dog, etc.), bacteria (yeast), and environmental samples (water from a paddy field, etc.). These are in the less frequently analyzed sample category in ThingMR. The samples in ThingMR had various APSRs and peak numbers (Figure 1, Supplementary Table S1). Rosemary (Lamiaceae) and Inuyomogi and Ryuno-giku (Asteraceae) had a large number of peaks and smaller APSRs, although many samples in these families are included in ThingMR. These species may biosynthesize many species-specific chemicals. When the distribution was viewed by categories, the plants were scattered widely. Foods contained a lower number of peaks and higher APSRs. This suggests that most of the chemicals we eat in foods are ubiquitous. The distribution shown in Figure 1 will change in future, as the number of samples increases. The samples in the category with lower APSRs should be actively analyzed to efficiently enhance the coverage of chemicals.

### Functions of XMRs

**Figure 2.**
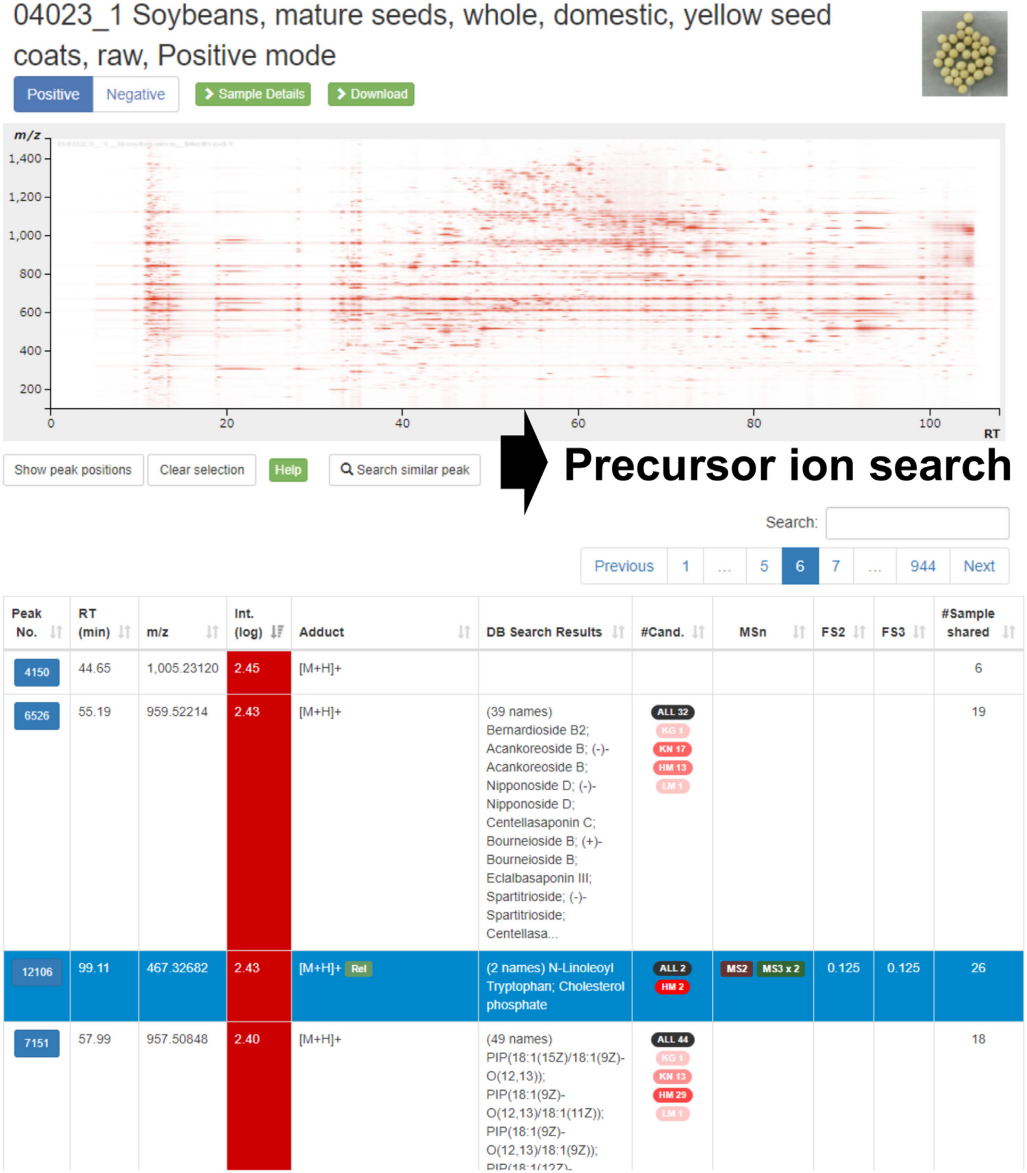
The peak list page of a sample in FoodMR. An example of the data obtained from soybean (Food ID: 04023_1) in ESI-positive mode is shown. The button with a peak ID at the left of the table row links to the detailed peak page (Figure 3). The ‘Search similar peak’ button performs the precursor ion search (Figure 4) using the *m/z* and retention time of the selected peak (the row highlighted in blue). The number in the ‘#Sample shared’ column represents the number of samples detected by the similar peak search with 5 ppm and 1 min tolerance for *m/z* value and retention time, respectively. Similar to the APSR in Figure 1, the number represents a sample specificity measure of the peak and facilitates searching sample (group)-specific peaks.

**Figure 3.**
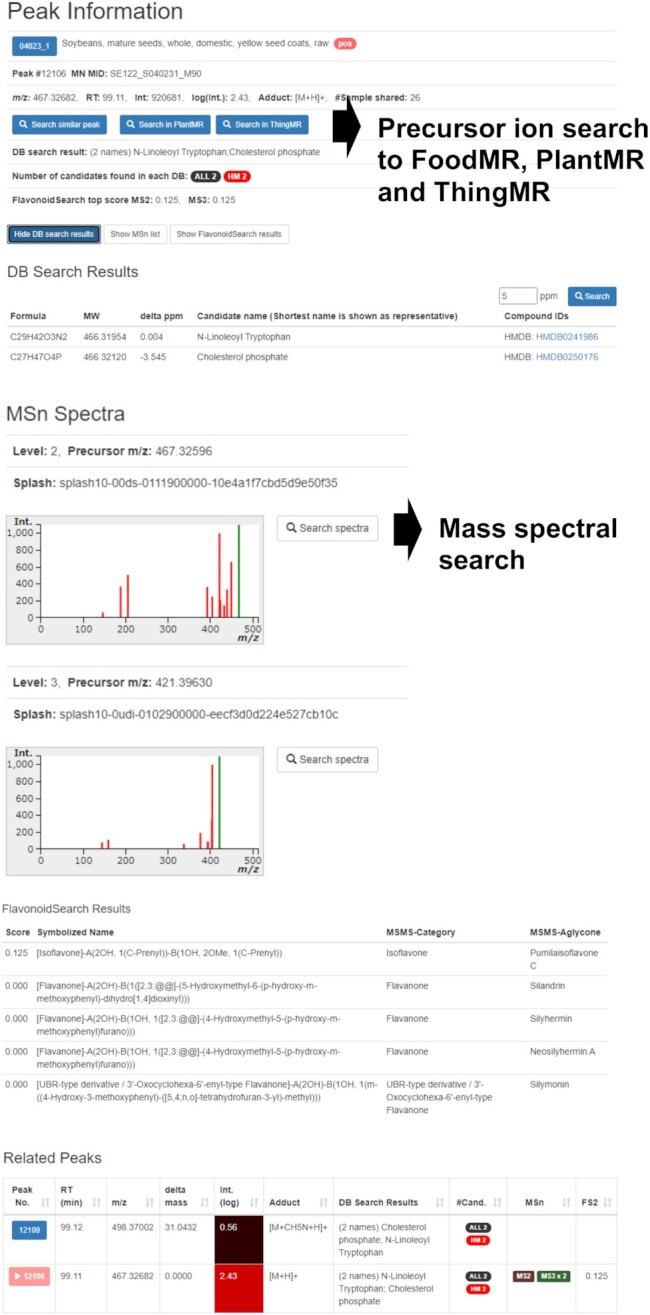
The detailed peak information page of FoodMR. The example of the peak ID 12106 detected in soybean (Food ID: 04023_1) in ESI-positive mode is shown. The buttons ‘Search similar peak’ and ‘Search in PlantMR’ buttons perform the precursor ion search (Figure 4) using the *m/z* value and retention time of the peak to FoodMR and PlantMR, respectively. The button ‘Search in ThingMR’ performs a similar peak search to ThingMR using the *m/z* value and approximate retention time. The ‘Search spectra’ buttons at the right of the mass spectral image perform a mass spectral search (Figure 5) using the spectra. The predicted flavonoid aglycones based on the spectrum and related peaks, such as different adduct ions, are also available.

This section briefly introduces the major functions of XMRs for obtaining insight into sample-specific localizations of the metabolites peaks, namely, search functions and APIs. XMRs also provide essential functions as web-based databases, such as browsing peak lists (Figure 2) and peak details (Figure 3) and downloading the raw and processed data files. In addition, the mass chromatogram data, presented in two-dimensional images, are available in Microsoft PowerPoint files named ‘MassChroBook.’ The two-dimensional pictures help to present intuitively the similarity/difference in the metabolic profiles that may be missed out by statistical methods such as multivariate analysis.

#### Peak search by precursor ion mass value and retention time

**Figure 4.**
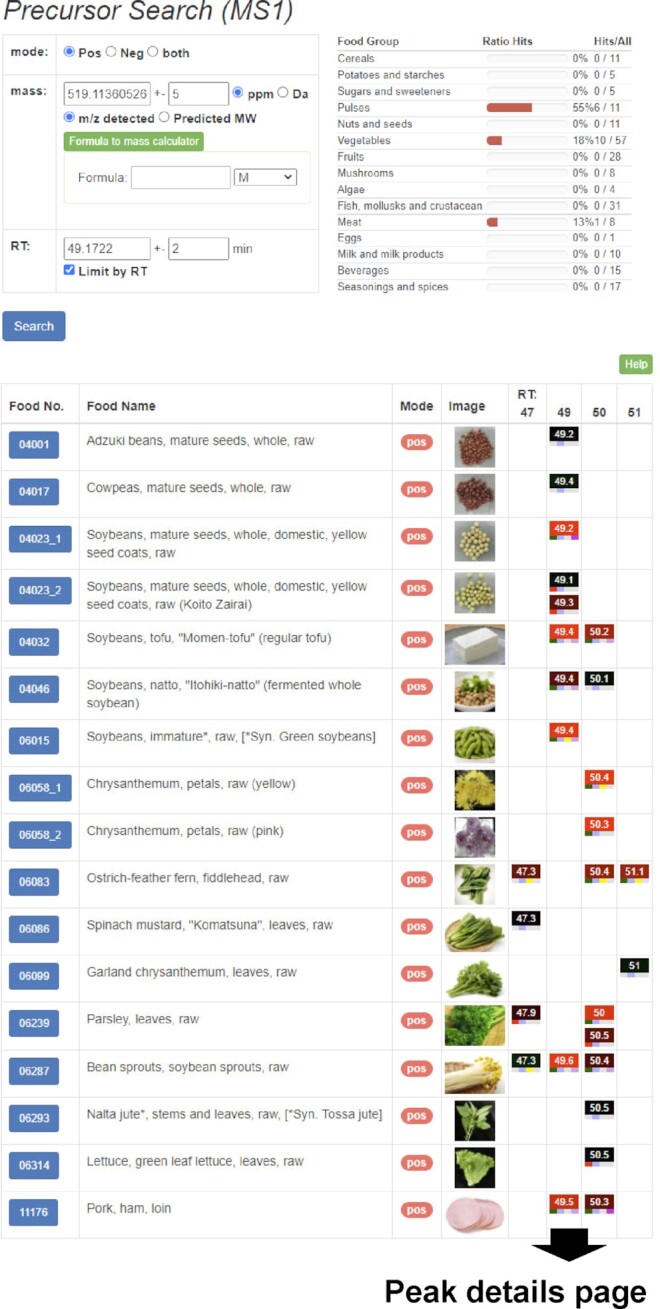
The precursor ion search function of FoodMR. The example of the results matching a peak detected with ESI-positive mode in soybean (*m/z* 519.1136, retention time 49.17 min) with given mass and retention time tolerances of 5 ppm and 2 min, respectively, is shown. The ratios of samples that contained the matched peaks per the samples in the category (food group) are summarized in the top-right. In the results table on the bottom, the matched peaks represented as peak icons (colored rectangles) are aligned by their nominal retention time. The peak icon summarizes the retention time, peak intensity, presence of MS^n^ spectral data, compound database search results, adduct ions, and FlavonoidSearch results. Details of the icons are available on the help page of FoodMR (http://metabolites.in/foods/about/help). The peak icon links to the details of the peak information (Figure 3).

Users can search peaks detected in the samples based on the precursor ions that match a given accurate mass value and a retention time of LC (Figure 4). The search result was represented as a table in which the rectangle icons for the matched peaks are arranged in the columns corresponding to their nominal retention time. The peak icon shows the peak intensity and retention time by the color and the number, respectively. This presentation of the results table helps users to grasp the sample specificity of the queried peak. The small rectangles at the bottom of the peak icon show the other characteristics of the peak, namely the availability of MS^n^ spectra, the availability of database search results, the type of adduct ion assigned, and the FlavonoidSearch score. These help users to check the appropriateness of adduct ion assignment, obtain the MS^*n*^ spectra when it lacks in the queried peak, and facilitate further annotation of the peak.

In the case of the peaks registered in XMRs, users can directly perform the search using the peak information by two procedures. One is selecting the row of the peak on the peak list of the sample and clicking the ‘Search similar peak’ button (Figure 2). The other is clicking the ‘Search similar peak’ button on the detailed peak information page (Figure 3). From the peak information page, users can search for similar peaks in the other XMR databases based on the *m/z* value and the approximated retention time (Figure 3).

More generally, as described below, in the case of the peaks measured by other LC–high resolution MS platforms or compounds of known formula, the users can obtain the potential counterparts in XMRs by not specifying the retention time in the search. The results show the candidate isomers and their sample specificity. The number of possible isomers and differences in their MS^*n*^ spectra will also help the prioritization and structural annotation of the unknown peak. Furthermore, users can identify the counterpart peak in XMRs if similar LC–MS conditions were applied in the user's platform by following these steps: analyzing the same or similar sample using the user's platform; identifying the commonly detected peaks in the platform and XMR; and calculating the equations to convert the retention time from one to the other based on, for example, the regression curve for the retention times of commonly detected peaks. As an example, we present the construction of a converter for the LC–MS systems used in a study in MetaboLights (MTBLS771) (24) (Supplementary Data 1). We have also provided an MS Excel file for the conversion between ThingMR and FoodMR/PlantMR on the download page. Therefore, we can generally use the peak information in XMRs as a reference for annotating unknown peaks detected by a wide range of LC–MS instruments.

The following given tolerances for mass value and retention time are recommended for searching: mass value, 5 ppm (FoodMR and PlantMR) and 20 ppm (ThingMR); and retention time, 1 min (FoodMR), 2 min (PlantMR) and 0.5 min (ThingMR) (Table 1). The actual mass accuracy of FT-ICR MS used in the FoodMR and PlantMR is less than 2 ppm in most cases. However, some peaks with higher intensity showed mass drift up to 5 ppm. Therefore, we recommend giving 5 ppm mass tolerances for arbitrary peak comparisons for FoodMR and PlantMR. The actual mass accuracy of Q-ToF used in the ThingMR is below 5 ppm in most cases. However, in some cases, such as peaks with lower intensity, the accuracy is not within 15 ppm. Therefore, we recommend allowing 20 ppm for ThingMR. The reproducibility of the retention time is usually <1% of the gradient time. Users can check the reproducibility using the standard chemical peaks available on the ‘Compound’ page in ThingMR or the internal standard peaks in the MassChroBook or mass chromatogram data.

#### Peak search by mass spectra

**Figure 5.**
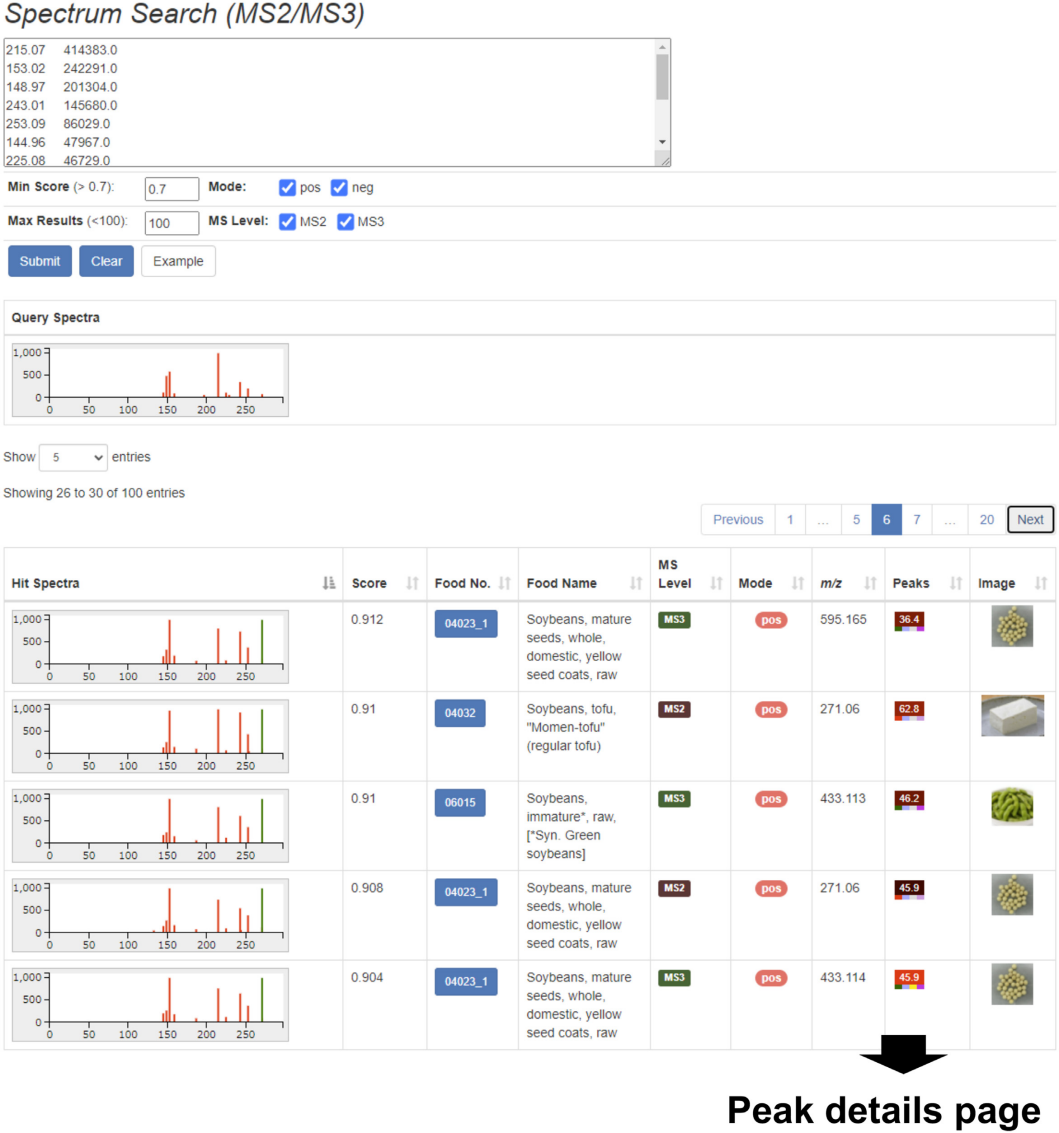
The mass spectra search function of FoodMR. The example of results obtained with one of MS^3^ spectra (precursor *m/z* 271.1) of a soybean peak (Food ID: 04023_1, Peak ID: 5065) detected in ESI-positive mode is shown. The queried mass spectrum and the mass spectra of matched peaks are represented by images (bar graphs). Both MS^2^ and MS^3^ match in this example. The peak icon links to the peak details page (Figure 3).

The cross-sample distribution of the peaks with similar mass spectra can be examined using the search functions based on the mass spectral similarity (Figure 5). Because DDA is applied for MS^*n*^ or MS/MS analysis in XMRs, fragmentation data are available for only a proportion of the peaks. Nevertheless, we can obtain the sample-specific localization of the peak by combining the mass spectral search results with the precursor search function mentioned above. This is one of the most significant differences between XMRs from the other databases solely based on mass spectra. By clicking the ‘Search spectra’ button on the detailed peak information page, users can immediately perform the search using the spectrum (Figure 3).

The search results are provided along with images of mass spectra to allow intuitive judgment of the identity/similarity of the spectra (Figure 5). The mass spectral similarity is estimated by the cosine product correlation coefficient. Because the similarity score depends on the number of product ions, we cannot determine a specific threshold value to judge the identity of the metabolites. Therefore, the XMRs provide images (bar graphs) of mass spectra in the results table (Figure 5). In the calculation of the similarity score, the mass spectral data were rounded to nominal mass values. The rounding was adopted because of the low mass accuracy of ion-trap MS (<0.5 Da) used in FoodMR and PlantMR and to accelerate the search rate.

It is unique for FoodMR and PlantMR that the MS^3^ spectra of the unknowns obtained from untargeted metabolome analysis are searchable among a wide variety of samples. We can search for candidates of compound derivatives that display the same fragmentation in MS^3^ as in the MS^2^ spectrum of the compound. This helps, for example, achieve a comprehensive search for potential glycosides of a known or unknown aglycone.

As a precaution for general use, please note that the compatibility of the mass spectra between platforms should be considered for a proper understanding of the search results. The mass spectrum of a compound obtained by linear-ion-trap MS in FoodMR and PlantMR differs from that obtained by Q-Tof MS in ThingMR. Similarly, the spectra in XMRs differ from the users’ own data. Therefore, when the cross-sample distribution of the peaks that have similar mass spectra is evaluated, users should seek a counterpart peak in the XMRs first and then, if found, perform the spectral search based on the counterpart peak. The mass spectral search of XMRs did not aim at searching for the own mass spectra of the users by themselves, as provided by MASST (10) and the mass spectral library MassBank (25).

#### APIs

It is a significant feature of XMRs that the untargeted metabolome data obtained from a wide range of samples are ready to use for bioinformatics via application programming interfaces (APIs). XMRs provide APIs in representational state transfer (REST) format for most of all the available functions on web browsers. Therefore, the users directly use these functions from the external computational programs. The APIs allow the users to perform the automatic and massive search of a large number of data and subsequent complex data analysis, which is not practical to perform only in web browsers. The search results of the APIs are available in JavaScript Object Notation (JSON) format, which is suitable for processing with computational programming languages such as Python. Sample program codes for the use of the APIs written in Python are available on the help page. Sample codes for searching candidates of novel flavonoids (described later) and searching peaks specifically accumulated in certain specific sample groups (e.g. helpful for biomarker discovery) have been currently provided (July 2022). A URL-based RESTful format as the API input is also advantageous for easy and precise recording and sharing of specific information in XMRs, search conditions, and so on. Examples are shown in the column ‘Link to the peak information in FoodMR’ in Table 3.

**Table 3.** Candidate novel flavonoids found by data mining in FoodMR

Food ID	Category	Name	Peak ID	Retention time (min)	*m/z* ([M + H]+)	FlavonoidSearch score (MS^3^)	Observation	Candidate substituent*	Link to the peak information in FoodMR
05014	Nuts and seeds	Walnuts, roasted	2560	40.0	785.0703	0.500	Walnuts specific		http://metabolites.in/foods/peak/05014/pos/2560
06058_2	Vegetables	Chrysanthemum, petals, raw (pink)	1704	37.8	553.1189	0.600	Present in pink but absent in yellow flowers of chrysanthemum	(Malonyl)-Galactosyl, (Malonyl)-Glucosyl, (Glucuronosyl)-Lactoyl	http://metabolites.in/foods/peak/06058_2/pos/1704
06267	Vegetables	Spinach, leaves, all season, raw	7956	60.8	683.1613	0.667	Spinach specific	(Rhamnosyl)-Galacturonosyl, ((OMe)-Rhamnosyl)-Glucosyl	http://metabolites.in/foods/peak/06267/pos/7956
06267	Vegetables	Spinach, leaves, all season, raw	8020	61.5	713.1721	0.667	Spinach specific	(Glucuronosyl)-Glucuronosyl	http://metabolites.in/foods/peak/06267/pos/8020
06314	Vegetables	Lettuce, green leaf lettuce, leaves, raw	1507	35.7	727.1357	0.650	Asteraceae specific	(Malonyl)-(Glucuronosyl)-Glucosyl	http://metabolites.in/foods/peak/06314/pos/1507
07078	Fruits	Citrus, ‘Sudachi’, peel, raw	5754	57.8	643.1668	0.500	‘Sudachi’ specific	Dihydrophaseoyl, (p-Hydroxybenzoyl)-Galactosyl, (*p*-Hydroxybenzoyl)-Glucosyl	http://metabolites.in/foods/peak/07078/pos/5754
03001	Sugars and sweeteners	Sugars, brown sugar lamp	4749	47.9	587.0706	0.800	A lot of peaks with the same MS^3^ spectra are found in rice leaves in PlantMR.	(Sulfo)-Glucuronosyl	http://metabolites.in/foods/peak/03001/pos/4749
03002	Sugars and sweeteners	Sugars, ‘Wasanbonto’ (traditional non-centrifugal soft white cane sugar)	2542	47.5	587.0699	0.667			
17001	Seasonings and spices	Japanese Worcester sauce, common type	2102	47.7	587.0708	0.667			
17031	Seasonings and spices	Seasoning sauce, oyster sauce	3085	47.8	587.0708	0.667			

*Known substituents were identified by FlavonoidSearch tool.

### Application of XMRs

#### An integrated analysis for discovery of metabolite and gene candidates in species-specific metabolic pathways

**Figure 6.**
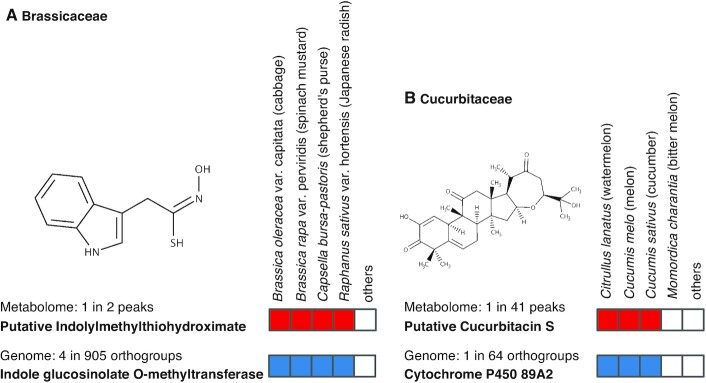
Metabolite and gene candidates found in the integrated metabolome and genome analysis. Using the peak table constructed from 535 samples in ThingMR (March 2022) and published genome data, the candidate metabolites and orthologs localized in specific plant species were found. The candidates putatively related to (**A**) glucosinolate and (**B**) cucurbitacin biosynthetic pathways and their cross-species distribution are shown.

Using the peak table constructed from 535 samples in ThingMR (March 2022) and the published genome data, we calculated the pairs of metabolite peaks and orthologs that are detected in specific biological classes (Figure 6). We found a metabolite peak (*m/z* 207.057 in positive mode, RT 10.67) that was specifically detected in *Brassica oleracea*, *Brassica rapa*, *Capsella bursa-pastoris* and *Raphanus sativus*, and not detected in other plant species. A candidate for this peak was an intermediate metabolite in the glucosinolate biosynthesis pathway, 3-indolylmethylthiohydroximate. We found 905 orthogoups that showed the same plant species-specificity; among them, four orthogroups were indole glucosinolate *O*-methyltransferases. The results suggest that this metabolome–genome integrated analysis facilitated the discovery of reasonable metabolite and gene pairs in species-specific metabolic pathways. Using the same approach, we found a pair of putative isomers of cucurbitacin S (*m/z* 481.29 in positive mode, RT 22.0 and 18.44) and an ortholog cytochrome P450 89A2 that were detected in Cucurbitaceae species, *Citrullus lanatus, Cucumis melo* and *Cucumis sativus*, but not in other plants. The detailed metabolic pathway for the biosynthesis of cucurbitacin S and the specific substrate of the cytochrome P450 89A2 are not fully understood. Therefore, the results provide a new working hypothesis for further identifying candidate metabolites and studying this pathway in depth. The precise annotation procedures for these metabolites are described in the Supplementary Methods. Briefly, the candidates were annotated using precursor ion mass and MS/MS spectra at the confidence level of 3 (putatively characterized compound classes) proposed by the Metabolomics Standards Initiative (MSI) (26,27).

#### Annotation of carpaine-related metabolites in papaya

The sample-specific localization of peaks in FoodMR supported annotation of the carpaine-related biosynthetic and/or degradation intermediates in papaya (28). Carpaine, found in papaya, is an alkaloid with antiviral and antiplasmodial activities (29), but the biosynthetic pathway of carpaine is not fully understood. Hiraga et al. (28) annotated eight carpaine-related metabolites, including carpaine, carpaic acid, and three novel putative structures in papaya fruits, based on their accurate mass values and MS/MS spectra obtained by LC–MS. They did not identify these metabolites, probably owing to the limited availability of their authentic standards. However, they showed strong support for the annotation using FoodMR in which untargeted metabolome data from mature papaya fruits are registered. The mass values of the eight carpaine-related metabolites, except two abundantly accumulated in immature fruits, were detected in the mature papaya fruits in FoodMR. Furthermore, the mass values of these were not detected in other 221 foods. As carpaine was found in specific plants, papaya and *Azima tetracantha* (30), these results strongly suggested that the annotated metabolites were carpaine derivatives. Using the APIs of FoodMR, we were able to find other candidate carpaine derivatives specific to papaya, e.g. a putative derivative of dehydrocarpamic acid (http://metabolites.in/foods/peak/07109/pos/2567) with the same MS^3^ spectrum as that of dehydrocarpamic acid. We annotated the candidate using precursor ion mass and MS^2^ and MS^3^ spectra at the MSI confidence level of 3 (putatively characterized compound classes) (Supplementary Methods). These candidates would be prioritized for further identification and functional estimation.

#### Identification of okaramines in the rhizosphere of hairy vetch

The accumulation of food metabolome data led us to the identification of non-food-derived compounds, okaramines. We identified pesticidal okaramines for the first time from nature (31). Okaramines were first identified in soybean pulp (okara) inoculated with *Penicillium simplicissimum* AK-40 (32) and were found to have insecticidal activity same as that of ivermectin (33). However, the presence of okaramines in nature is unknown. We found several candidates of okaramine species in the rhizosphere soil of the manure plant hairy vetch (*Vicia villosa* Rotch subsp. *villosa*) in a metabolome analysis of soil samples. The MS^*n*^ spectra showed good agreement with their chemical structures. In addition, we found that no peaks matched the okaramines of the 969 000 peaks from 222 foods (ESI-positive mode) registered in FoodMR. The absence in general foods was reasonable when assuming the candidate peaks were okaramines that were probably derived from soil bacteria in the rhizosphere. This was a strong driver for us to identify okaramines using authentic standards. Finally, we identified okaramine A, B and C at the MSI confidence level of 1 (identified compounds) by the accurate precursor ion mass, retention time, and MS^2^ and MS/MS spectra (Supplementary Methods).

#### Discovery of novel flavonoid candidates

By data mining using the APIs of XMR, we found several candidates for novel flavonoids in this study. The peaks in FoodMR that matched the following conditions are potential candidates of novel flavonoid derivatives: (i) no results in the compound database search; (ii) MS^3^ spectra and (iii) a high similarity score from FlavonoidSearch for MS^3^ spectrum (see Supplementary Methods). A search with multiple conditions like this case is not practical for manual operation in a web browser. Using APIs, we can perform complex searches easily and within a short time. Through a search of approximately 969 000 peaks in FoodMR (ESI-positive mode), we found 23 peaks that matched the conditions above in 10 min. Then, we manually checked the sample specificity and features of the putative substituents using the FoodMR website. We found some interesting candidates, such as those detected in specific foods and those with neutral loss masses that have not been well reported previously (Table 3). Although the candidates were not identified using authentic standards (annotated at the MSI confidence level of 3 ‘putatively characterized compound classes,’ see Supplementary Methods), they might be targets in a future study. The Python program code used for this search is available on the help page of FoodMR as an example of the use of APIs.

During this investigation, we noticed that sample specificity in non-food data helped to annotate the peaks in foods. Among the 23 peaks, a single candidate was hard to annotate by its sample specificity in FoodMR. The peak with *m/z* 587.07 was specifically detected in the food category ‘Sugars and sweeteners’ and foods in ‘Seasonings and spices,’ which includes sugars. The peak was present in brown sugar lumps and ‘Wasanbonto’ (traditional non-centrifugal sugar), but absent in granulated sugar. The key to annotating the situation was determined by PlantMR. We found many peaks in rice leaves with the same MS^3^ spectra as that of the unannotated peak. This information reminded us that both brown sugar lump and ‘Wasanbonto’ are made from sugar cane. Therefore, the peak in the sugars might be a flavonoid with an aglycone actively biosynthesized in Poaceae.

#### Detection of caffeine in honey

We found a peak annotated as caffeine—a plant-derived alkaloid established to accumulate in coffee and tea—was also detected in honey in FoodMR. We identified the peak in ThingMR as caffeine using an authentic standard compound at the MSI confidence level of 1 (identified compounds) based on the identical accurate mass value of the precursor ion, the retention time confirmed by co-injection, and MS/MS spectra (Supplementary Methods). We found that the presence of caffeine depends on the honey products; namely four out of seven honey products contained caffeine. This observation was in good agreement with previous reports. Some citrus plants accumulate caffeine in the flowers and nectar, and caffeine can be found in honey (34). Moreover, rewarding honeybees with caffeine enhanced their memory of the floral scent (35). Therefore, caffeine production in the flower is understood as a strategy for increasing reproductive benefits by enhancing the pollinator's fidelity (35). Although this case was a rediscovery of previous knowledge on the known compound caffeine, is also showed that the comparison of the large variety of samples with XMRs could lead to the construction of new and proper working hypotheses without any prerequisite knowledge in a specific research field.

### Precautions for data interpretation and use

We should draw users’ attention to precautions to avoid mis- and over-interpretation of XMR results, although some of them are mentioned elsewhere in this study. In addition, when using the XMR results for further establishment of working hypotheses and investigation, it is always necessary for users to confirm the results. First, the sample specificity results from the precursor ion mass search depend on the samples deposited at the time of the search. Please consult the kind of samples (presence or absence) using the sample list for appropriate interpretation. Moreover, sample specificity results based solely on the mass spectral search are further dependent on the coverage of mass spectra obtained through DDA. Combined use of the precursor search would be required for proper interpretation (see the section ‘Peak search by mass spectra’). Second, peak quality should be considered. Please note that a significant number of metabolites are undetectable under the LC–MS conditions used in XMRs. For example, as shown on the ‘Compound’ page, only 6 out of 20 amino acids were separated, ionized, and detected in ThingMR. Caffeine was only detected by the ESI-positive mode. The unique ‘not detected’ information for examined authentic standards on the ‘Compound’ page would help users in speculating the detectable metabolites based on their hydrophobicity, mass values, etc. The similarity of the precursor ion mass, retention time, and MS/MS spectra to those of authentic standards strongly suggests that the peak is the standard compound. However, the peak could still not be the standard but a rather similar isomer that cannot be separated and distinguished under our conditions. At the very least, our system cannot distinguish between most of stereoisomers. Third, peak quantity (intensity) is neither an absolute nor accurate value because it depends on various factors such as the amount of sample injected, metabolite extraction and ionization efficiency, ion suppression or enhancement, detector sensitivity at the time of analysis, and peak signal distribution (for the log-transformed intensity centered by the median). Finally, more general remarks should be made. The peak data in XMRs contain potential false positives and false negatives. Failures in estimating precursor ion mass could also occur as a result of monoisotopic ion peak mischaracterization. This case is more likely to occur in the case of a higher mass value and a multivalent peak with a low-signal intensity for the monoisotopic ion. Misestimation of adduct ions is also possible. The alignment results on the download page may contain misalignments. The database search results and FlavonoidSearch results only provide candidates and do not guarantee the presence or absence of the compounds. These possibilities must be carefully examined during further investigations, especially for peaks annotated solely by precursor ion mass values.

## DISCUSSION

Comparable untargeted metabolome data, which have been lacking in systems biology and are now provided by XMRs, are a good resource for further data-driven research into unknown chemicals based on their sample-specific localizations. As exemplified in the integrated analysis of the metabolome and the genome, the data resource facilitates the use of metabolome data in multi-omics studies. In the example of papaya and okaramines, FoodMR provided appropriateness for the presence of both the unknown unknowns (truly novel compounds) and known unknowns (known compounds not described in the sample) (36), respectively, in the specific samples. Using caffeine as an example, the comparison of the various samples that are not compared in a general design of a single study would find an unexpected occurrence of metabolites that can lead to new working hypotheses. In the example of the data mining for novel flavonoids, the APIs of XMRs were used as a powerful bioinformatics tool for the top-down discovery of unknowns based on their sample specificity. Although the sample specificity-based discovery of unknown metabolites has been reported using GC–MS-based database (5), as far as we know, XMRs is the first public databases in which LC–MS-based untargeted metabolome data are comparable based on the precursor ion mass, retention time, and MS^n^ or MS/MS spectra in total 984 various samples. The provision of such datasets will strongly promote further use of the metabolome data, for example, correlation analysis of metabolites and genes and the discovery of unknown metabolites for quality control markers of specific organisms, among others.

Information on the precursor ion rather than the MS/MS or MS^*n*^ spectra is useful in a public resource for depicting the sample-specific localization of the metabolites. MS/MS or MS^*n*^ spectra are often used for comparisons of structural identity and the prediction of the structures of the metabolites (3,37). However, the data are dependent on the instruments and collision energy conditions; hence, the interpretation and confidence of the results remain under discussion. In contrast, the accurate mass value of the precursor ions is robustly obtained with commonly used high-resolution MS. As exemplified here, the papaya-specific localization of the carpaine-related candidates was successfully examined based on the accurate mass values obtained by a different MS platform from that used in FoodMR (28). Thus, the use of precursor ions is advantageous for enlarging the comparison spaces of the metabolites. We also previously demonstrated the usefulness of the accurate mass records (AMRs) for the annotation of unknown metabolites and pointed out the limited availability of AMRs in the public domain as an issue for promoting the annotation (7). XMRs provide more than 11 107 619 AMRs obtained from 984 samples in total (Table 2, July 2022). The availability of these data on the XMR website and via APIs should promote the discovery, annotation, and identification of unknowns.

To assist further valuable knowledge discoveries, the concept of XMR should be expanded, and some issues should be solved, as discussed below.

### Expansion of the concept of XMR

The concept of XMR should be expanded through the following four aspects.

#### Variety of the samples

The variety of the samples should be increased. As exemplified in the discovery of novel flavonoids, the non-food data obtained from plant samples helped to annotate the unknown metabolites found in foods. Conversely, the okaramine cases showed that food data helped to identify the metabolites derived from non-food samples. These results obtained by the previously unexpected specific comparisons suggest that increasing the sample varieties synergistically accelerates the annotation of the unknowns based on their sample-specific localizations. Therefore, not only the samples in specific categories (such as foods and plants) should be measured, but also any other samples (e.g. from animals, bacteria, the environment, waste, artificial products, historical samples) can be added into a dataset for comparison. Consequently, we are now expanding ThingMR. As demonstrated in Supplementary Figure S1, the unique peaks have not yet been saturated. The index APSR (Figure 1) we proposed in this article would facilitate selecting the category of the unanalyzed samples.

#### Coverage of the metabolites

The coverage of metabolites should also be expanded by the addition of several robust instrumental platforms. Only a single reversed-phase LC method was used for the construction of XMRs. Therefore, a large portion of the metabolites, such as highly hydrophilic compounds (e.g. sugars, organic acids and most amino acids), highly hydrophobic compounds (e.g. carotenoids and non-polar lipids), and volatiles (e.g. low-molecular terpenes, aldehydes, and alcohols) are omitted and not compared in the current XMRs. The use of other separation technologies for constructing them, such as lipid-focused LC–MS or supercritical fluid chromatography–MS, hydrophilic interaction chromatography (HILIC)–MS for hydrophilic compounds, GC–MS for volatiles, and CE–MS for ionic water-soluble compounds, and the establishment of robust measurement procedures for arbitrary comparison of a large number of samples are required. Also, the mass accuracy and coverage of MS/MS spectra should be improved using high-spec MS instruments. Some widely targeted approaches will facilitate the annotation of the data. Furthermore, any other compound detection technologies, such as chemical sensors, may be applicable as long as they are reproducible and robust for large-scale comparison. The application of multiple platforms to the same sample set should strongly enhance the annotation and discovery of novel metabolites. For example, the combined use of LC–MS and GC–MS for various samples would be helpful in discovering the volatiles and their water-soluble glycosides as storage forms by correlation analysis of their sample specificity.

#### Number of comparable datasets

Not only our XMRs, but also other comparable datasets should be constructed in different countries. For the efficient enlargement of the abovementioned points (sample variety and metabolite coverage), the specification of a base center that can actively produce high-quality and comparable data is ideal, for two main reasons. First, a general metabolome analysis service where samples are provided by the researchers is not suitable because the sample diversity is affected by researchers’ interests, causing it to be biased toward some specific samples, such as humans and model organisms. For example, out of 128 156 data entries measured by reversed-phase LC–MS published from MetaboLights (January 2022), 50.9%, and 16.1% were samples from humans and perennial ryegrass, respectively; only 767 unique organism parts were present. The active collection and analysis of unanalyzed samples are required to enlarge the sample variety efficiently. Second, a general analysis service/center where the analysis methods are customized according to the users’ requests is not suitable to maintain sufficient data quality. The restricted use of the analytical instruments in constant conditions with a single or several specific method(s) is required for the production of robust and comparable untargeted metabolome data. This machine use policy is especially important for producing high-quality data using multiple platforms for expanding metabolite coverage. However, it is not practical to establish a centralized base that analyses all ‘things’ from all over the world owing to the maintenance costs, throughput bottlenecks, and need for international shipping of the materials. Therefore, multiple datasets in which the sample data are comparable in the dataset should be constructed by individual base centers established in various countries. As exemplified here, even a single dataset contributes to knowledge discovery. Furthermore, some datasets can be compared based on the similarity of the analytical methods, as demonstrated in the comparisons between FoodMR and ThingMR or data from MetaboLights and XMRs (Supplementary Data 1). Data obtained from a common sample readily available in every country, such as a major crop, could be used to link and standardize the datasets.

#### Bioinformatic researchers

An increase in the number of bioinformatic researchers who discover new knowledge and working hypotheses and add value to the database is essential for expanding the concept of XMR. We expect that the number of bioinformatic researchers who are interested in XMRs will increase as the abovementioned points occurs. The provision of APIs, which enables the integration of metabolome data to other studies, such as genome, transcriptome, proteome, and phenome data, is critical for the bioinformatic use of XMR. For this purpose, further enrichment of sample metadata, such as biological taxonomy, treatment, and processing, as well as provision of the metadata described with proper ontology and controlled vocabulary as a machine-readable format should be promoted in the future (see the next section). Broadening the research backgrounds of these bioinformatic researchers will help to achieve a multifaceted analysis of untargeted metabolome data and lead to discoveries of unexpected, new knowledge and hypotheses.

### Further improvements

#### Enrichment of sample metadata

For further data analysis based on metadata, such as that performed with ReDU (12) and foodMASST (11,13), and for appropriate interpretation of the results, the sample metadata needs to be enriched. In FoodMR, we adopted the sample names, sample IDs, and category names that were defined in the Standard Tables of Food Composition in Japan-2015 (Seventh Revised Version). For PlantMR and ThingMR, however, we assigned simple category names that we defined ourselves and did not actively work on assigning ontology and using controlled vocabulary, although the taxonomy IDs from NCBI were available for the samples in ThingMR. This policy is because of two reasons. First, the required ontology depends on the users’ purposes. The use of ThingMR is diverse because it contains data from various samples. Therefore, enrichment of the metadata would begin when the specific target is focused on by the users’ community in particular study fields. Second, the attachment of ontology would be a bottleneck in data publication. Attaching proper ontology and controlled vocabulary to the sample metadata requires a deep understanding of the definition of the classes and vocabulary. Improper attachment and bias of the completeness of the work among the samples could lead to misinterpretation of the results. For these reasons, we provide simple category information, and currently, the annotation of metadata is left to the user. However, the next enhancement in XMRs would be the attachment of rich metadata like the ReDU project of GNPS (12). Any support from the research community for assigning appropriate and confidential metadata is helpful. We would certainly bridge questions from the users about the metadata of particular samples to the person responsible for those samples.

#### Confidence in the peak data

Confidence in the peak data should be improved, especially at the qualitative level. XMR contains many latent false-positive and false-negative peaks arising from both the analytical method and data processing. Most of the false positives are possibly derived from the data processing. In XMR, we adjusted the peak detection parameters of the processing to pick as many peaks as possible with lower intensities, which improves the assignment of adduct ions and the discrimination of isotopic peaks. However, although this increases the false-positive rate, this is not considered a major drawback to the estimation of the sample specificity of the unknown peaks because the sample specificity should also be considered from the peak intensities. In contrast, a major cause of the false negatives is considered to be the analytical method, namely, the application of a single method for metabolite extraction and the limitation of the injected sample amount on the LC to avoid carryovers. This issue can likely be overcome by the application of additional analytical procedures with different extraction methods, as mentioned in *Expansion of metabolite coverage*. Future increases in the analytical repetition of similar samples will contribute to minimizing the false positives and false negatives.

The annotation of the artificial peaks and provision of such information should be improved in the future. It is known that a significant number of peaks detected by untargeted LC–MS are those of derivative ions derived from the same compounds, such as various adduct ions, multimers, and in-source fragments; thus, the real number of unique compounds in a sample is much smaller than the observed number of peak (38–40). In the current XMR, we provide all peak data, including such derivative ion peaks, because their information may, in some cases, help with metabolite annotation. For example, in the tomato data in ThingMR, a peak of tomatidine is detected as derivative ion peaks as an in-source dissociation of a glycoside, putative tomatine. As the free form of tomatidine is not detected in the same sample, the fragmented peak helped to annotate tomatine based on the MS/MS spectra. In the current XMRs, the variations in the adduct ions and the dissociations of well-known small molecules, such as water, are provided on the peak details page (Figure 3). Possible in-source fragment peaks should be provided in future improvements.

There is no efficient way to avoid the false-negative/positive peaks or under-/overestimations of the peak intensity caused by ion suppression/enhancement by the co-eluted compound while using the current LC–MS conditions. This issue could be overcome by the application of other ionization principles; one example would be probe electrospray ionization (PESI), which is known to be tolerant to ion suppression (41).

Quantitativity of the metabolites should be considered in the future. For the current purpose of XMR, the quantitation accuracy of the detected peaks is not crucial for the discovery and prioritizing the unknowns because the sample-specific accumulations should be evaluated in the subsequent detailed investigations. However, the addition of precisely identified and quantified data definitely improves the value of data mining through the datasets. Therefore, the application of (widely-) targeted metabolome data should be added in the future.

#### Identification of the unknowns

The acceleration of metabolite identification is the ultimate issue in the study of unknown metabolites. After focusing on the unknowns for further detailed investigation using XMRs, they must be identified. The sample-specific distribution provided by XMRs is helpful in determining the samples that abundantly accumulate the unknown metabolites for purification and structural elucidation. When combined with recently progressing technologies such as the crystalline sponge method (42), microcrystal electron diffraction (MicroED) (43,44), and a synthesis-based reverse-metabolomic approach (45), it is a prospective strategy for accelerating the identification of unknown metabolites.

## DATA AVAILABILITY

PowerGetBatch, MassChroViewer, and FlavonoidSearch are academic-free software tools available from the KOMICS website (https://www.kazusa.or.jp/komics).

PowerGetBatch: (https://www.kazusa.or.jp/komics/software/PowerGetBatch).

FlavonoidSearch: (https://www.kazusa.or.jp/komics/software/FlavonoidSearch).

MassChroViewer: (https://www.kazusa.or.jp/komics/software/MassChroViewer).

The experimental metadata of XMRs have been deposited in Metabolonote (http://metabolonote.kazusa.or.jp/) (16) under the accession IDs as follows: FoodMR, SE112-123, SE169-172; PlantMR, SE61, SE198-205; and ThingMR, SE221-226 (July 2022).

## Supplementary Material

gkac1058_Supplemental_Files

## References

[1] Djoumbou-Feunang Y. , Pon A., Karu N., Zheng J., Li C., Arndt D., Gautam M., Allen F., Wishart D.S. CFM-ID 3.0: significantly improved ESI-MS/MS prediction and compound identification. Metabolites. 2019; 9:72. 31013937 10.3390/metabo9040072 PMC6523630

[2] Schymanski E.L. , Ruttkies C., Krauss M., Brouard C., Kind T., Dührkop K., Allen F., Vaniya A., Verdegem D., Böcker S. et al. Critical assessment of small molecule identification 2016: automated methods. J. Cheminform. 2017; 9:22. 29086042 10.1186/s13321-017-0207-1 PMC5368104

[3] Wang M. , Carver J.J., Phelan V.V., Sanchez L.M., Garg N., Peng Y., Nguyen D.D., Watrous J., Kapono C.A., Luzzatto-Knaan T. et al. Sharing and community curation of mass spectrometry data with global natural products social molecular networking. Nat. Biotechnol. 2016; 34:828–837. 27504778 10.1038/nbt.3597 PMC5321674

[4] Tsugawa H. Advances in computational metabolomics and databases deepen the understanding of metabolisms. Curr. Opin. Biotechnol. 2018; 54:10–17. 29413746 10.1016/j.copbio.2018.01.008

[5] Lai Z. , Tsugawa H., Wohlgemuth G., Mehta S., Mueller M., Zheng Y., Ogiwara A., Meissen J., Showalter M., Takeuchi K. et al. Identifying metabolites by integrating metabolome databases with mass spectrometry cheminformatics. Nat. Methods. 2018; 15:53–56. 29176591 10.1038/nmeth.4512 PMC6358022

[6] Olivon F. , Allard P.M., Koval A., Righi D., Genta-Jouve G., Neyts J., Apel C., Pannecouque C., Nothias L.F., Cachet X. et al. Bioactive natural products prioritization using massive Multi-informational molecular networks. ACS Chem. Biol. 2017; 12:2644–2651. 28829118 10.1021/acschembio.7b00413

[7] Ara T. , Sakurai N., Takahashi S., Waki N., Suganuma H., Aizawa K., Matsumura Y., Kawada T., Shibata D TOMATOMET: a metabolome database consists of 7118 accurate mass values detected in mature fruits of 25 tomato cultivars. Plant Direct. 2021; 5:e00318. 33969254 10.1002/pld3.318 PMC8082711

[8] Haug K. , Cochrane K., Nainala V.C., Williams M., Chang J., Jayaseelan K.V., O’Donovan C MetaboLights: a resource evolving in response to the needs of its scientific community. Nucleic Acids Res. 2020; 48:D440–D444. 31691833 10.1093/nar/gkz1019 PMC7145518

[9] Sud M. , Fahy E., Cotter D., Azam K., Vadivelu I., Burant C., Edison A., Fiehn O., Higashi R., Nair K.S. et al. Metabolomics workbench: an international repository for metabolomics data and metadata, metabolite standards, protocols, tutorials and training, and analysis tools. Nucleic Acids Res. 2016; 44:D463–D470. 26467476 10.1093/nar/gkv1042 PMC4702780

[10] Wang M. , Jarmusch A.K., Vargas F., Aksenov A.A., Gauglitz J.M., Weldon K., Petras D., da Silva R., Quinn R., Melnik A.V. et al. Mass spectrometry searches using MASST. Nat. Biotechnol. 2020; 38:23–26. 31894142 10.1038/s41587-019-0375-9 PMC7236533

[11] West K.A. , Schmid R., Gauglitz J.M., Wang M., Dorrestein P.C. foodMASST a mass spectrometry search tool for foods and beverages. NPJ Sci. Food. 2022; 6:22. 35444218 10.1038/s41538-022-00137-3 PMC9021190

[12] Jarmusch A.K. , Wang M., Aceves C.M., Advani R.S., Aguirre S., Aksenov A.A., Aleti G., Aron A.T., Bauermeister A., Bolleddu S. et al. ReDU: a framework to find and reanalyze public mass spectrometry data. Nat. Methods. 2020; 17:901–904. 32807955 10.1038/s41592-020-0916-7 PMC7968862

[13] Gauglitz J.M. , West K.A., Bittremieux W., Williams C.L., Weldon K.C., Panitchpakdi M., Di Ottavio F., Aceves C.M., Brown E., Sikora N.C. et al. Enhancing untargeted metabolomics using metadata-based source annotation. Nat. Biotechnol. 2022; 10.1038/s41587-022-01368-1. PMC10277029 35798960

[14] Sakurai N. , Shibata D Tools and databases for an integrated metabolite annotation environment for liquid chromatography-mass spectrometry-based untargeted metabolomics. Carot. Sci. 2017; 22:16–22.

[15] Sakurai N. , Ara T., Enomoto M., Motegi T., Morishita Y., Kurabayashi A., Iijima Y., Ogata Y., Nakajima D., Suzuki H. et al. Tools and databases of the KOMICS web portal for preprocessing, mining, and dissemination of metabolomics data. BioMed Res. Int. 2014; 2014:194812. 24949426 10.1155/2014/194812 PMC4052814

[16] Ara T. , Enomoto M., Arita M., Ikeda C., Kera K., Yamada M., Nishioka T., Ikeda T., Nihei Y., Shibata D. et al. Metabolonote: a wiki-based database for managing hierarchical metadata of metabolome analyses. Front. Bioeng. Biotechnol. 2015; 3:38. 25905099 10.3389/fbioe.2015.00038 PMC4388006

[17] Emms D.M. , Kelly S. OrthoFinder: phylogenetic orthology inference for comparative genomics. Genome Biol. 2019; 20:238. 31727128 10.1186/s13059-019-1832-y PMC6857279

[18] Kanehisa M. , Sato Y., Kawashima M., Furumichi M., Tanabe M. KEGG as a reference resource for gene and protein annotation. Nucleic Acids Res. 2016; 44:D457–D462. 26476454 10.1093/nar/gkv1070 PMC4702792

[19] Afendi F.M. , Okada T., Yamazaki M., Hirai-Morita A., Nakamura Y., Nakamura K., Ikeda S., Takahashi H., Altaf-Ul-Amin M., Darusman L.K. et al. KNApSAcK family databases: integrated metabolite-plant species databases for multifaceted plant research. Plant Cell Physiol. 2012; 53:e1. 22123792 10.1093/pcp/pcr165

[20] Wishart D.S. , Jewison T., Guo A.C., Wilson M., Knox C., Liu Y., Djoumbou Y., Mandal R., Aziat F., Dong E. et al. HMDB 3.0–The human metabolome database in 2013. Nucleic Acids Res. 2013; 41:D801–D807. 23161693 10.1093/nar/gks1065 PMC3531200

[21] Fahy E. , Subramaniam S., Murphy R.C., Nishijima M., Raetz C.R., Shimizu T., Spener F., van Meer G., Wakelam M.J., Dennis E.A. Update of the LIPID MAPS comprehensive classification system for lipids. J. Lipid Res. 2009; 50(Suppl):S9–S14. 19098281 10.1194/jlr.R800095-JLR200 PMC2674711

[22] Akimoto N. , Ara T., Nakajima D., Suda K., Ikeda C., Takahashi S., Muneto R., Yamada M., Suzuki H., Shibata D. et al. FlavonoidSearch: a system for comprehensive flavonoid annotation by mass spectrometry. Sci. Rep. 2017; 7:1243. 28455528 10.1038/s41598-017-01390-3 PMC5430893

[23] Gachon C.M. , Langlois-Meurinne M., Saindrenan P. Plant secondary metabolism glycosyltransferases: the emerging functional analysis. Trends Plant Sci. 2005; 10:542–549. 16214386 10.1016/j.tplants.2005.09.007

[24] Delaporte G. , Cladière M., Camel V. Untargeted food chemical safety assessment: a proof-of-concept on two analytical platforms and contamination scenarios of tea. Food Control. 2019; 98:510–519.

[25] Horai H. , Arita M., Kanaya S., Nihei Y., Ikeda T., Suwa K., Ojima Y., Tanaka K., Tanaka S., Aoshima K. et al. MassBank: a public repository for sharing mass spectral data for life sciences. J. Mass Spectrometry: JMS. 2010; 45:703–714. 20623627 10.1002/jms.1777

[26] Sumner L. , Amberg A., Barrett D., Beale M., Beger R., Daykin C., Fan T.M., Fiehn O., Goodacre R., Griffin J. et al. Proposed minimum reporting standards for chemical analysis. Metabolomics. 2007; 3:211–221. 24039616 10.1007/s11306-007-0082-2 PMC3772505

[27] Blaženović I. , Kind T., Ji J., Fiehn O. Software tools and approaches for compound identification of LC-MS/MS data in metabolomics. Metabolites. 2018; 8:31. 29748461 10.3390/metabo8020031 PMC6027441

[28] Hiraga Y. , Ara T., Sato N., Akimoto N., Sugiyama K., Suzuki H., Kera K. Metabolic analysis of unripe papaya (*Carica papaya* L.) to promote its utilization as a functional food. Biosci. Biotechnol. Biochem. 2021; 85:1194–1204. 33704369 10.1093/bbb/zbab014

[29] Radhakrishnan N. , Lam K.W., Norhaizan M.E. Molecular docking analysis of *carica**papaya* linn constituents as antiviral agent. Int. Food Res. J. 2017; 24:1819–1825.

[30] Bennett R.N. , Mellon F.A., Rosa E.A., Perkins L., Kroon P.A. Profiling glucosinolates, flavonoids, alkaloids, and other secondary metabolites in tissues of *azima**tetracantha* l. (Salvadoraceae). J. Agric. Food Chem. 2004; 52:5856–5862. 15366832 10.1021/jf040091+

[31] Sakurai N. , Mardani-Korrani H., Nakayasu M., Matsuda K., Ochiai K., Kobayashi M., Tahara Y., Onodera T., Aoki Y., Motobayashi T. et al. Metabolome analysis identified okaramines in the soybean rhizosphere as a legacy of hairy vetch. Front. Genet. 2020; 11:114. 32153648 10.3389/fgene.2020.00114 PMC7049541

[32] Hayashi H. , Takiuchi K., Murao S., Arai M. Structure and insecticidal activity of new indole alkaloids, okaramines a and b, from *penicillium**simplicissimum* AK-40. Agric. Biol. Chem. 2014; 53:461–469.

[33] Furutani S. , Nakatani Y., Miura Y., Ihara M., Kai K., Hayashi H., Matsuda K. GluCl a target of indole alkaloid okaramines: a 25 year enigma solved. Sci. Rep. 2014; 4:6190. 25155752 10.1038/srep06190 PMC4143795

[34] Kretschmar J.A. , Baumann T.W. Caffeine in *citrus* flowers. Phytochem. 1999; 52:19–23.

[35] Wright G.A. , Baker D.D., Palmer M.J., Stabler D., Mustard J.A., Power E.F., Borland A.M., Stevenson P.C. Caffeine in floral nectar enhances a pollinator's memory of reward. Science. 2013; 339:1202–1204. 23471406 10.1126/science.1228806 PMC4521368

[36] Wishart D.S. Computational strategies for metabolite identification in metabolomics. Bioanalysis. 2009; 1:1579–1596. 21083105 10.4155/bio.09.138

[37] Kind T. , Tsugawa H., Cajka T., Ma Y., Lai Z., Mehta S.S., Wohlgemuth G., Barupal D.K., Showalter M.R., Arita M. et al. Identification of small molecules using accurate mass MS/MS search. Mass Spectrom. Rev. 2018; 37:513–532. 28436590 10.1002/mas.21535 PMC8106966

[38] Brown M. , Wedge D.C., Goodacre R., Kell D.B., Baker P.N., Kenny L.C., Mamas M.A., Neyses L., Dunn W.B. Automated workflows for accurate mass-based putative metabolite identification in LC/MS-derived metabolomic datasets. Bioinformatics. 2011; 27:1108–1112. 21325300 10.1093/bioinformatics/btr079 PMC3709197

[39] Jankevics A. , Merlo M.E., de Vries M., Vonk R.J., Takano E., Breitling R. Separating the wheat from the chaff: a prioritisation pipeline for the analysis of metabolomics datasets. Metabolomics. 2012; 8:29–36. 22593722 10.1007/s11306-011-0341-0 PMC3337394

[40] Mahieu N.G. , Patti G.J. Systems-Level annotation of a metabolomics data set reduces 25000 features to fewer than 1000 unique metabolites. Anal. Chem. 2017; 89:10397–10406. 28914531 10.1021/acs.analchem.7b02380 PMC6427824

[41] Hiraoka K. , Ariyada O., Usmanov D.T., Chen L.C., Ninomiya S., Yoshimura K., Takeda S., Yu Z., Mandal M.K., Wada H. et al. Probe electrospray ionization (PESI) and its modified versions: dipping PESI (dPESI), sheath-flow PESI (sfPESI) and adjustable sfPESI (ad-sfPESI). Mass Spectrom. (Tokyo). 2020; 9:A0092. 33299735 10.5702/massspectrometry.A0092 PMC7708747

[42] Hoshino M. , Khutia A., Xing H., Inokuma Y., Fujita M. The crystalline sponge method updated. IUCrJ. 2016; 3:139–151. 27006777 10.1107/S2052252515024379 PMC4775162

[43] Ito S. , White F.J., Okunishi E., Aoyama Y., Yamano A., Sato H., Ferrara J.D., Jasnowski M., Meyer M. Structure determination of small molecule compounds by an electron diffractometer for 3D ED/MicroED. CrystEngComm. 2021; 23:8622–8630.

[44] Jones C.G. , Martynowycz M.W., Hattne J., Fulton T.J., Stoltz B.M., Rodriguez J.A., Nelson H.M., Gonen T. The CryoEM method MicroED as a powerful tool for small molecule structure determination. ACS Cent. Sci. 2018; 4:1587–1592. 30555912 10.1021/acscentsci.8b00760 PMC6276044

[45] Dorrestein P. , Gentry E., Collins S., Panitchpakdi M., Belda-Ferre P., Stewart A., Wang M., Jarmusch A., Avila-Pacheco J., Plichta D. et al. A Synthesis-Based Reverse Metabolomics Approach for the Discovery of Chemical Structures from Humans and Animals. 2021; Research Square doi:30 August 2021, preprint: not peer reviewed https://doi.org/10.21203/rs.3.rs-820302/v1.

