# Supplementary material for: The Thing Metabolome Repository family (XMRs): comparable untargeted metabolome databases for analyzing sample-specific unknown metabolites
Source: Nucleic Acids Res. 2022 Nov 24;51(D1):D660–77. doi: 10.1093/nar/gkac1058 (PMC9825447; doi:10.1093/nar/gkac1058)
Supplement: gkac1058_Supplemental_Files [file gkac1058_supplemental_files.zip › Supplementary.r4_sub.pdf]

The Thing Metabolome Repository family (XMRs): comparable untargeted metabolome databases for analyzing sample-specific unknown metabolites

Nozomu Sakurai[1,2,3,†,*], Shinichi Yamazaki[4,†], Kunihiro Suda[2], Ai Hosoki[2], Nayumi Akimoto[2], Haruya Takahashi[5], Daisuke Shibata[2] and Yuichi Aoki[4,6,*]

[1] Bioinformation and DDBJ Center, National Institute of Genetics, 1111 Yata, Mishima, Shizuoka 411-8540, Japan

[2] Kazusa DNA Research Institute, 2-6-7 Kazusa-kamatari, Kisarazu, Chiba 292-0818, Japan

[3] Sakura Scientific Co. Ltd., 35 Horinouchi, Odawara, Kanagawa 250-0853, Japan

[4] Tohoku Medical Megabank Organization, Tohoku University, 2-1 Seiryo-machi, Aoba-ku, Sendai, Miyagi 980-8573, Japan

[5] Division of Food Science and Biotechnology, Graduate School of Agriculture, Kyoto University, Gokasho, Uji, Kyoto 611-0011, Japan

[6] Graduate School of Information Sciences, Tohoku University, 6-3-09 Aramaki-Aza-Aoba, Aoba-ku, Sendai, Miyagi 980-8679, Japan

* To whom correspondence should be addressed. Tel: +81-55-981-6895; Fax: +81-55-981-9448; Email: sakurai@nig.ac.jp, ns@sakura-kagaku.com

Correspondence may also be addressed to YA. Tel: +81-22-274-6040; Fax: +81-22-274-6040; Email: yuichi.aoki.e3@tohoku.ac.jp

† These authors are equally contributed.

SUPPLEMENTARY DATA

Contents:

Supplementary Methods

Supplementary Figures 1 and 2

Supplementary Methods

Sample preparation

The details of the samples and injected amount of the samples for LC–MS analysis are described on the Metabolonote website (http://metabolonote.kazusa.or.jp/) (1). For FoodMR, 222 food items commonly available at the local markets in Japan were selected from the approximately 2,200 food items listed in the Standard Tables of Food Composition in Japan -2015 (Seventh Revised Version) published by the Ministry of Education, Culture, Sports, Science and Technology, Japan (https://www.mext.go.jp/en/policy/science_technology/policy/title01/detail01/1374030.htm). Based on the Standard Tables, parts of the foods, such as the eggshell of raw egg, were discarded. For PlantMR, 28 plant-related samples, including model plants, cultured cells, and culture medium, were used. For ThingMR, more than 734 samples, including animals, plants, bacteria, foods, traditional Kampo medicine, environmental samples, commercial products, model organisms, and authentic standard compounds, were used. The solid samples were frozen in liquid nitrogen and homogenized in liquid nitrogen to a fine powder using a mortar and pestle. The sample was mixed with 3 volumes (w/v or v/v) of 100% methanol solution, 40 volumes of 75% methanol solution, or 100 volumes of 75% methanol solution, depending on the water content of the sample. The methanol solutions contained 25 μM (for FoodMR and PlantMR) or 1 μM (for ThingMR) of 7-hydroxy-5-methylflavone as an internal standard (IS). The sample-methanol solution in a 2 mL tube was homogenized with a zirconia bead (5 mm diameter) using a Mixer Mill MM 300 (for FoodMR and PlantMR, QIAGEN K.K.) or MM 400 (for ThingMR, Verder Scientific, Co. Ltd) at 25 Hz for 2 × 2 min. The homogenate was centrifuged at 17,400 ×*g*, 5 min at 4°C. For FoodMR and PlantMR, a supernatant was passed through a polytetrafluoroethylene filter (Millex LG, pore size 0.2 μm, Millipore) and applied to a C18 silica column (MonoSpin C18, GL Sciences Inc.) for removing highly hydrophobic compounds. For ThingMR, the supernatant was first applied to a C18 silica column (MonoSpin C18, GL Sciences Inc.) and then passed through a polytetrafluoroethylene filter (Millex LG, pore size 0.2 μm, Millipore). The filtrate was used for LC–MS analyses. The same extraction procedure with 75% methanol containing IS was performed without a sample to prepare mock samples, which were used as negative controls.

LC–MS analysis

Detailed conditions of the LC–MS analysis are also described on the help page of XMRs and the Metabolonote website.

- For FoodMR and PlantMR

An LC separation with a longer elution time for obtaining as many as MS^2 and MS^3 spectra by data-dependent acquisition (DDA) and high-resolution mass spectrometry for measuring accurate mass values of precursor ions were conducted. The Agilent 1100 system (Agilent) and Finnigan LTQ-FT (Thermo Fisher Scientific) were used. An aliquot (20 μL) of the methanol extract was applied to a TSKgel ODS-100V column (4.6 mm × 250 mm, 5 μm, Tosoh Corporation) connected after a guard column (TSKgel guardgel ODS-100V, 4.6 mm, 5 μm, Tosoh Corporation), and separated by water containing 0.1% (v/v) formic acid (Solvent A) and acetonitrile containing 0.1% (v/v) formic acid (Solvent B). The gradient program was as follows: 3% B (0 min), 97% B (90 min), 97% B (100 min), 3% B (100.1 min), and 3% B (107 min). The flow rate was set at 0.25 mL/min for 0–100 min and 0.5 mL/min for 100.1–107 min. The column oven temperature was set to 40°C. To monitor the HPLC eluate, a photodiode array detector was used with a wavelength range of 200–650 nm. The compounds separated by the LC were detected by MS with electrospray ionization (ESI) in positive or negative mode and the conditions are shown in Supplementary Table 2. In FoodMR, Method 1 and Method 5 for positive mode and Method 1 for negative mode were applied. In PlantMR, the combinations of Methods 1–7 and ionization polarity were different between the samples, but at least one method for accurate mass measurement of the precursor ion (Methods 1–4) was applied. Other MS setting parameters were as follows: spray voltage, 4.0 kV; capillary temperature, 300°C; nitrogen sheath gas, 40 arbitrary units; and auxiliary gas, 15 arbitrary units. The raw data were obtained by Xcalibur software (ver. 2.0.7, Thermo Fisher Scientific).

- ## For ThingMR

Nexera X2 system (Shimadzu Corporation) and Compact system (Bruker Japan K.K.) were used. An aliquot (2 μL) of the methanol extract was applied to an InertSustain AQ-C18 column (2.1 mm × 150 mm, 3 μm, GL Sciences) connected after a guard column (InertSustain AQ-C18 Cartridge Guard Column E, GL Sciences), and separated by water containing 0.1% (v/v) formic acid (Solvent A) and acetonitrile (Solvent B). The gradient program was as follows: 2% B (0 min), 2 % B (3 min), 98% B (30 min), 98% B (35 min), 2% B (35.1 min), and 2% B (42 min). The flow rate was set at 0.2 mL/min. The column oven temperature was set to 40°C. The compounds separated by LC were detected using the two MS conditions below. 1) Conditions for ESI-positive mode. The following MS settings were used: Ionization, ESI; Polarity, positive; Data acquisition, Profile mode; Scan rate, 1 Hz; Mas scan range, 50-1200; End plate offset, 500 V; Capillary voltage, 4000 V; Nebulizer gas (N2) pressure, 2.5 Bar; Dry gas (N2) flow, 8.0 L/min; Dry gas temperature, 200°C; Transfer Funnel1 RF, 200.0 Vpp; Funnel2 RF, 200.0 Vpp; In-source CID Energy, 0.0 eV; Hexapole RF, 50.0 Vpp; Quadrupole Ion Energy, 3.0 eV; Low Mass m/z, 55.00; Collision energy, 10.0 eV; Collision RF, 450.0 Vpp; Transfer Time, 80.0 us; and PrePulse Storage, 3.0 us. The following MS/MS settings were used: Auto MS/MS, on; Precursor ion number, 5; Isolation width, 3-15 Da; Collision energy, 35

eV; Active Exclusion, on; Exclude, after 3 spectra; Release, after 0.3 min; Reconsider Precursor, on; and if Current intens. / Previous Intens., 2.0. 2) Conditions for ESI-negative mode. The MS settings were the same as those in the ESI-positive mode, with the following exceptions: Polarity, negative; and MS/MS Collision energy, 30 eV. For calibrating the mass values, 1 mM sodium formate in 50% (v/v) 2-propanol was injected directly into the MS at 38.5–40.5 min of LC separation with a flow rate of 0.1 mL/min. The eluent at 0–3 min was wasted. The raw data were obtained by HyStar software (ver. 3.2 SR4, Bruker Daltonik, GmbH).

- ### Quality control

IS was used for maintaining the quality of the reproducibility of the retention time and the mass accuracy of each analyzed data. The IS retention time was controlled at 73 ± 1 and 21.5 ± 0.15 min for Food/PlantMR and ThingMR, respectively. The mass accuracy was controlled at ±1 and ±10 ppm for Food/PlantMR and ThingMR, respectively. The average values and standard deviations of the retention time and mass accuracy of IS peaks are presented in Supplementary Table 5.

Data processing

- ### Mass calibration

In ThingMR, the mass values of the detected ions in the raw data were calibrated using the signals of sodium formate detected at 39–42 min using Compass DataAnalysis software (ver. 4.2, Bruker Daltonik, GmbH).

- ### Peak detection and alignment

The raw data (FoodMR and PlantMR) and calibrated raw data (ThingMR) were converted to mzXML-formatted files using the MSConvert function of the ProteoWizard software (ver. 3.0, https://proteowizard.sourceforge.io/) (2). The PowerGetBatch software (https://www.kazusa.or.jp/komics/software/PowerGetBatch) (3,4) was used for peak detection, deisotoping, adduct ion assignment, peak alignment, and compound database search. The parameter setting files for PowerGetBatch used were available on the Download page of the XMR websites.

For FoodMR, three different sets of peak detection parameters were used for a single sample data obtained with Method 1 and a single set of parameters for the sample data obtained with Method 5 (Supplementary Table 2). A single set of parameters was applied for the mock data.

For PlantMR, in the case of the sample with more than three biological or technical replications obtained with high-resolution settings (Methods 1–4, Supplementary Table 2), a single set of peak

detection parameters was applied for peak detection. In the case of samples without replications obtained in Methods 1–4, three sets of parameters were applied to a single sample data. A single set of parameters were applied to the data obtained by low-resolution settings (Methods 5 or 7, Supplementary Table 2). A single set of parameters was applied to the mock data.

For ThingMR, three different sets of peak detection parameters were applied for each sample, and a single set of parameters was used for the mock sample.

The peaks detected in each polarity of the sample data and mock data measured in the same experimental batch were aligned. The peaks detected with all results from the sample data obtained at high resolution and not detected in the mock data were selected as valid peaks. For PlantMR, valid peaks were further selected manually using Microsoft Excel with consideration of the analytical replications of the sample. For ThingMR, the peaks with a retention time of less than 3 min or greater than 32 min were omitted. The most intense and major pattern of a single MS^2 (FoodMR and PlantMR) or MS/MS (ThingMR) and up to two MS^3 (FoodMR and PlantMR, positive mode) spectra in the alignment results were selected as representative mass spectra for each peak.

- ## Database searching

A compound database search was performed using the estimated accurate mass value and the assigned adduct ion of the peak. The UC2 database in the MFSearcher web service (http://webs2.kazusa.or.jp/mfsearcher/) (5) was used for a rapid cross-database search and compiling the constitutional isomers in one record. The following compound databases were used: KEGG (https://www.genome.jp/kegg/) (6); KNApSAcK (http://www.knapsackfamily.com/KNApSAcK_Family/) (7); Human Metabolome Database (HMDB, https://hmdb.ca/) (8); LIPID MAPS (https://www.lipidmaps.org/) (9); and the flavonoid database in metabolomics.jp (http://metabolomics.jp/wiki/Category:FL). The mass tolerances of 5 ppm for FoodMR and PlantMR and 20 ppm for ThingMR were applied to the search.

- ## Prediction of flavonoid aglycones

The prediction of flavonoid aglycones was performed using the FlavonoidSearch tool (https://www.kazusa.or.jp/komics/software/FlavonoidSearch) (10) using the MS^n or MS/MS spectra obtained in positive mode.

Construction of the peak table

The peak table, comprising the valid peaks detected in 535 samples in ThingMR (March 2022), was

constructed using the alignment function in the PowerGetBatch software run on the NIG supercomputer. The alignment parameter setting files are available in the peak table data provided on the download page of ThingMR. The outline of the alignment algorithm and setting parameters are described below:

While processing a small number of samples, the following process is performed with alignment.method = 0. The parameters margin_rt_ref = 5 (seconds) and margin_rt_target = 5 (seconds) for retention time tolerance, factor_fwhm = 3 (times), min_fwhm = 3 (seconds), and max_fwhm = 15 (seconds) for evaluating the overlap of peaks based on the full width at half maximum of the peak, and margin_mass_resolution_low = 15 (ppm) for *m/z* tolerance were specified for finding the two-way best matched peaks between two sample data. Then, the peak groups connected by the best-match peaks in all samples were calculated. In this step, some peaks were removed from the peak groups (ungrouped peaks). Next, the ungrouped peaks were added to the groups using the following parameters: restore_unused_peaks.margin_rt_sec_find_unused_peak = 30.0 (seconds) and restore_unused_peaks.margin_mass_ppm_find_unused_peak = 10.0 (ppm) for detecting unused peaks and restore_unused_peaks.margin_rt_sec_find_used_peak = 10.0 (seconds) and restore_unused_peaks.margin_mass_ppm_find_used_peak = 5.0 (ppm) for detecting the peaks already added to the group.

In this study, the above-mentioned process was replaced with the following for the alignment of a large number of samples. Using the same parameter settings except for alignment.method = 1, the set of the first peak groups calculated between the first and second samples was used for the construction of the second peak groups with the third sample. Then, the procedure was repeated until the last sample. Thereafter, using the average values of retention time and *m/z* of the resulting peak groups as a reference, the peaks of each sample were allocated again to get the final set of the peak groups.

Finally, the peaks in the peak groups with similar retention time and *m/z* value specified by the parameters check_neighbor_alignments.margin_rt_sec = 30.0 (seconds) and check_neighbor_alignments.margin_mass_ppm = 5.0 (ppm), respectively, were revised and allocated again in proper peak groups by considering the peak intensity and retention time order. In these procedures, retention time drift between two sample data was considered based on the best-match peaks. The peak table (alignment result) is the output of the resulting peak groups.

Data mining for novel flavonoid candidates

Candidate novel flavonoids were searched using APIs of FoodMR. The peaks that matched all of the

following three conditions were selected: 1) had no results in the compound database search using KEGG (6), KNApSAcK (7), HMDB (8), LIPID MAPS (9), and a flavonoid database (http://metabolomics.jp/wiki/Category:FL) by a given mass tolerance at 5 ppm (see the section on "Database search"); 2) had MS^3 spectra; 3) had a similarity score estimated by FlavnoidSearch (10) using the MS^3 spectra of > 0.5. The program code (in the Python language) available on the help page of FoodMR (http://metabolites.in/foods/about/api) was used. The program was executed by Python 3.9.0 on a Windows 10 PC connected to the internet. The sample specificity of the resulting peaks that matched the conditions was checked on the FoodMR website using a web browser. The accurate neutral loss mass value was estimated by subtracting the accurate mass value of the peak and the theoretical mass value of the aglycone predicted by FlavonoidSearch. The substituents reported as known flavonoids (Supplementary Table 8 in (10)) that matched the neutral loss mass value were selected as candidate substituents.

Statictics for MetaboLights

The data files in ISA-Tab format (11) of all studies were downloaded from the public FTP archive of MetaboLights using the Wget tool on a Linux PC (January 2022). The published studies were verified by accessing the MetaboLights website with the study IDs. Then, all the assays were listed with the associated information as follows: Study Assay Technology Type, Characteristics[Organism], Characteristics[Organism part], Parameter Value[Instrument], Parameter Value[Chromatography Instrument], Parameter Value[Ion source], and Parameter Value[Mass analyzer]. The assays measured using LC–MS were filtered by the following conditions: assays that were measured using "mass spectrometry" as instrument type; any chromatography except containing the word "CE"; and "electrospray ionization" as ionization method. The selected assays were counted per the organism and organism part. The records with blank or the following values as Characteristics[Organism] were omitted from the count: #reference compound; #reference standards; acetonitrile; blank; blank sample; blank value; control; injection control; pbs buffer; pooled quality control; pooled sample; qc; qc_pool; quality control; quality control sample; reference compound; reference compound mix; reference sample; reference standards; sample preparation blank; simulated data; solvent; and solvent blank. The listing and selection above were processed using Excel software (Microsoft) and an in-house Java program.

Metabolite annotation and identification

The detailed procedures of identification and annotation of the metabolites exemplified in the "Application of XMRs" section are described below.

- An integrated analysis for discovery of metabolite and gene candidates in species-specific metabolic pathways

3-Indolylmethylthiohydroximate

We first verified the appropriateness of the assignment of adduct ions and that there was no possibility of in-source fragmentation of the detected peak using the mzXML-converted mass chromatogram data and MassChroViewer software. We found no candidates in the MASST database (12) for the representative peak detected in cabbage (http://metabolites.in/things/peak/224111/pos/828) nor an available authentic standard for identification. Therefore, we annotated the metabolite peak based on the possible fragmentation of the candidates. The compound database search (see above) found four candidates: 2-[(2-Furanylmethyl)thio]-6-methylpyrazine (HMDB: HMDB0036187), 2-Methyl-3 or 5 or 6-(furfurylthio)pyrazine (mixture of isomers) (HMDB: HMDB0032414), CBS 113A (KEGG: C14081), and 3-Indolylmethylthiohydroximate (KEGG: C16516 etc.). Among them, the chemical structure of 3-Indolylmethylthiohydroximate was reasonable for the observed fragment ions, as described below.

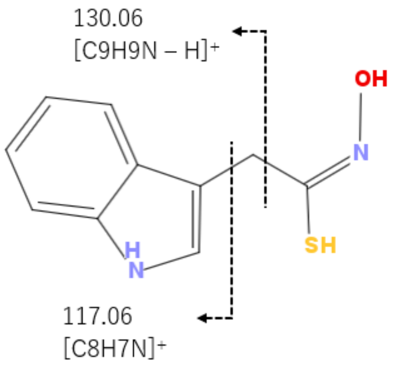

The structure of 3-Indolylmethylthiohydroximate and potential cleavages for the product ion mass values.

The structure prediction tool MS-FINDER (ver.3.52) (13,14) predicted the 3-Indolylmethylthiohydroximate as the top candidate. The default settings of MS-FINDER were used, except for setting 20 ppm for mass tolerances for MS1 and MS2 in the Basic settings. From these results, we annotated the peak as 3-Indolylmethylthiohydroximate at the confidence level of 3 (putatively characterized compound classes) proposed by the Metabolomics Standards Initiative (MSI) (15,16).

Cucurbitacin S

The appropriateness of the adduct ion assignment and fragmentation was verified as described above.

The representative peak detected at a retention time of 22 min in the melon root (http://metabolites.in/things/peak/224026/pos/4728) was assigned as $[M\text{-}H_2O+H]^+$, and the putative $[M+H]^+$ peak of the metabolite (http://metabolites.in/things/peak/224026/pos/4723) was confirmed exhibiting a similar species-specific distribution. The compound database search results showed 11 candidates. We found no matched spectra from authentic standards in MASST. The MS-FINDER tool predicted cucurbitacin S as the top candidate (20 ppm mass tolerance was set for MS1 and MS2). Although the MS/MS spectra of cucurbitacin S were not available, those with multiple fragment ions agreed with those from plant steroids. The putative isomer peak ($[M\text{-}H_2O+H]^+$) was detected at 18 min, and its $[M+H]^+$ peak showed similar MS/MS spectra and MS-FINDER results. Considering the plant species specificity, we annotated those peaks as putative cucurbitacin S and its isomer at the MSI confidence level of 3 (putatively characterized compound classes) (15,16).

- ## Annotation of carpaine-related metabolites in papaya

The carpaine-related metabolites were annotated by Hiraga et al. (17) based on the precursor ion mass and MS/MS spectra. FoodMR was used to confirm the papaya-specific distribution of these peaks and to enhance the confidence in their conclusion.

Among the six carpaine derivatives (Compounds 3–8) that Hiraga et al. (17) found in the mature papaya data in FoodMR, we confirmed that five (indicated below) displayed MS^2 spectra and essentially the same features as those reported by them (17).

Compound 3: http://metabolites.in/foods/peak/07109/pos/2810

Compound 4: http://metabolites.in/foods/peak/07109/pos/2585

Compound 5: http://metabolites.in/foods/peak/07109/pos/2787

Compound 7: http://metabolites.in/foods/peak/07109/pos/2398

Compound 8: http://metabolites.in/foods/peak/07109/pos/2123

Therefore, we annotated these five peaks as the carpaine derivatives at the MSI confidence level of 3 (putatively characterized compound classes) (15,16).

We found a candidate derivative of dehydrocarpamic acid (http://metabolites.in/foods/peak/07109/pos/2567) using the APIs of FoodMR. The MS^3 spectrum of the candidate was essentially the same as the MS^2 spectrum of putative dehydrocarpamic acid (Compound 4). Because the dehydrocarpamic acid had not been identified using authentic standards yet, we annotated the peak as a putative derivative of dehydrocarpamic acid at the MSI confidence of level 3 (putatively characterized compound classes) (15,16).

● Identification of okaramines in the rhizosphere of hairy vetch

As described in our previous study (18), okaramine A, B, and C were identified at the MSI confidence level of 1 (identified compounds) (15,16) using the authentic standards of okaramines based on the identity of the accurate mass value of the precursor ion, the retention time, and MS^2 and MS/MS spectra. Please see our previous study (18) for the details of MS^n and MS/MS annotations and identification.

● Discovery of novel flavonoid candidates

As described in the main text, novel flavonoid candidates were found based on their precursor ion mass and MS^3 spectra. Because the aglycones of these candidates have not been identified yet using authentic standards, we annotated them at the MSI confidence level of 3 (putatively characterized compound classes) (15,16).

● Detection of caffeine in honey

We noticed a peak equivalent to that of caffeine in honey using the precursor search function of FoodMR (http://metabolites.in/foods/search/precursor/mz/195.08765204510004/5/ppm/pos). The precursor ion mass, retention time, and features of MS^2 and MS^3 spectra were essentially similar to the peaks detected in coffee.

A peak in honey: http://metabolites.in/foods/peak/03022/pos/3027

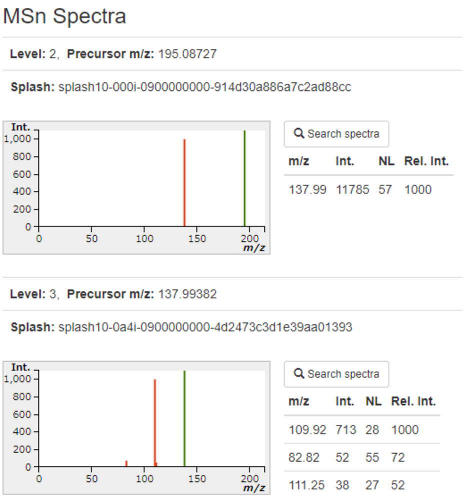

A peak in coffee: http://metabolites.in/foods/peak/16045/pos/2668

MSn Spectra

Level: 2, **Precursor m/z:** 195.08752

Splash: splash10-000i-0900000000-4998748b54137cc8be90

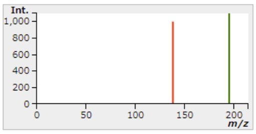

Search spectra

m/z	Int.	NL	Rel. Int.
137.96	4959548	57	1000

Level: 3, **Precursor m/z:** 137.95143

Splash: splash10-0a4i-0900000000-7ca4bdb3706cdc84bf6f

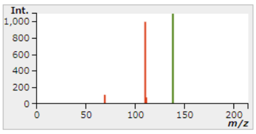

Search spectra

m/z	Int.	NL	Rel. Int.
109.88	255426	28	1000
68.88	28212	69	110
110.96	19939	27	78

These fragmentation features agreed with the data deposited in the MassBank database (19) as follows:

ID: MSBNK-Eawag-EA030309

Caffeine; LC-ESI-ITFT; MS2; CE: 30%; R=15000; [M+H]+

http://www.massbank.jp/RecordDisplay?id=MSBNK-Eawag-EA030309&dsn=Eawag

ID: MSBNK-Eawag-EA030310

Caffeine; LC-ESI-ITFT; MS2; CE: 45%; R=15000; [M+H]+

http://www.massbank.jp/RecordDisplay?id=MSBNK-Eawag-EA030310&dsn=Eawag

However, we could not identify these peaks in FoodMR using authentic standards before discarding the LC–MS instrument.

We identified the caffeine peaks in honey and other samples in ThingMR using the authentic standard (Tokyo Chemical Industry Col., Ltd.). The features of the precursor ion mass, the retention time, and MS/MS spectra were essentially the same as described below.

The peak for caffeine standard:

http://metabolites.in/things/peak/222020/pos/797

MSn Spectra

Level: 2, **Precursor m/z:** 195.08666

Splash: splash10-000i-0900000000-3cb4a6df5e70bdf3765f

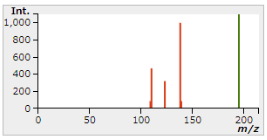

Search spectra

m/z	Int.	NL	Rel. Int.
138.06	25782	57	1000
110.07	12102	85	469
123.04	8262	72	320
139.07	2230	56	86
109.04	2210	86	85
195.09	1488	0	57

The candidate peak in Honey 4 (Sample ID: 223014):

http://metabolites.in/things/peak/223014/pos/1982

MSn Spectra

Level: 2, **Precursor m/z:** 195.08545

Splash: splash10-000i-0900000000-13004ffbcc538779fb79

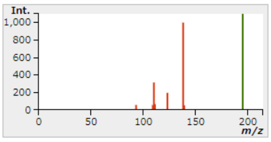

Search spectra

m/z	Int.	NL	Rel. Int.
138.06	2922	57	1000
110.07	918	85	314
123.04	574	72	196
195.09	246	-0	84
111.07	196	84	67
93.04	164	102	56
109.04	162	86	55
139.07	156	56	53
111.05	148	84	50

Precursor search results:

http://metabolites.in/things/search/precursor/mz/195.0856212427556/20/ppm/pos/11.9066/0.5

MS/MS spectral search results:

http://metabolites.in/things/search/spectrum/138.06,25782:110.07,12102:123.04,8262:139.07,2230:109.04,2210:195.09,1488

Furthermore, we observed a single peak for caffeine when the extracts from Honey 4 (Sample ID: 223014) and the caffeine standard were co-injected (data not shown). Therefore, we identified these honey peaks as caffeine at the MSI confidence level of 1 (identified compounds) (15,16).

Retention time conversion between the other LC–MS systems

To illustrate the retention time conversion between the data obtained by other LC–MS systems and

that in XMRs, we prepared a converter in the form of an MS Excel file (Supplementary Data 1). We used the data from a study (MTBLS771) (20) published by MetaboLights (21).

https://www.ebi.ac.uk/metabolights/MTBLS771

The untargeted metabolome data were obtained by LC–ToF–MS and LC–Orbitrap–MS under chromatographic conditions similar to those used in XMRs. We downloaded the following mass chromatogram data for green tea Samples 01 and 07.

LC–ToF: POS_ToF_GreenTea_S01n01.mzXML, POS_ToF_GreenTea_S07n01.mzXML

LC–Orbitrap: POS_Orbitrap_GreenTea_S01n01.mzML, POS_Orbitrap_GreenTea_S07n01.mzML

The metabolite peaks commonly detected in green tea samples from ThingMR (ID: 224095) and FoodMR (ID: 16037) were selected, and the counterpart peaks with similar precursor ion mass values were further selected from the mass chromatogram data of MTBLS771 using MassChroViewer (the "Common peak" sheet in the Supplementary Data 1). The peaks detected in all four mass chromatogram data from MTBLS771 were adopted, and the data from Sample 01 was recorded (the data from Sample 07 were used for checking the reproducibility of peak detection). Then, the slopes and intercepts of regression lines for the retention time of these peaks were calculated from the data (the "Regression line" sheet in Supplementary Data 1). Finally, using the slopes and intercepts, we provided the conversion functions for all combinations of the data (the "Converter" sheet).

Supplementary Figures

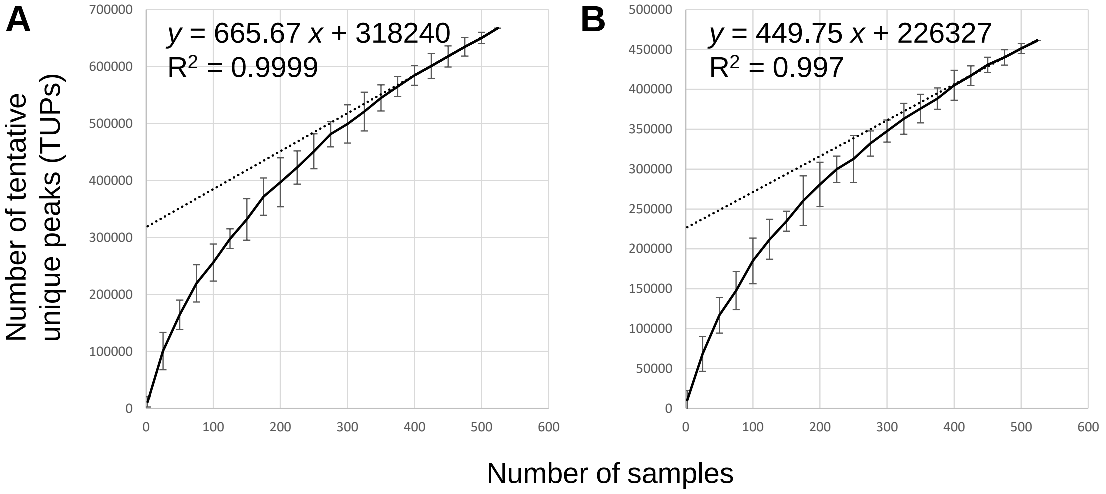

Supplementary Figure 1 Increase in tentative unique peaks as the number of samples increases.

The average number of tentative unique peaks (TUPs; the same or similar peaks aligned between the samples) calculated by 10 trials of randomly selected samples among 524 samples in ThingMR (March 2022) (except for standard chemicals) is shown for (A) ESI-positive mode and (B) ESI-negative mode. Only the TUPs assigned as $[M+H]^+$ for ESI-positive mode and $[M-H]^-$ for ESI-negative mode were used. The dotted lines are regression lines calculated using the six data points obtained from more than 400 samples. The function and R^2 value of the regression lines are presented as formulas. Error bars are SD.

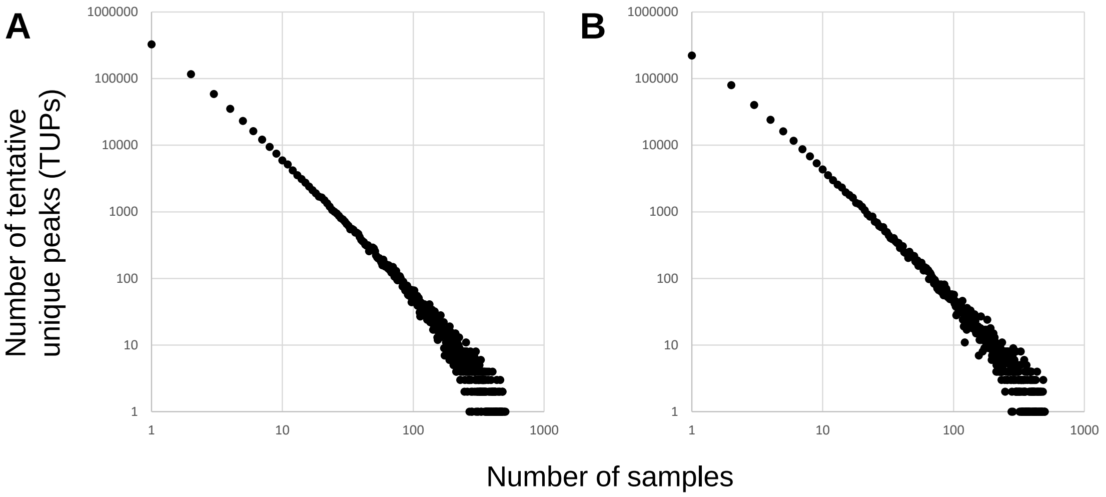

Supplementary Figure 2 Distribution of the number of tentative unique peaks commonly detected among the samples. (A) ESI-positive mode. (B) ESI-negative mode. The tentative unique peaks (TUPs; the same or similar peaks aligned between the samples) assigned as $[M+H]^+$ for ESI-positive mode and $[M-H]^-$ for ESI-negative mode calculated among 524 samples in ThingMR (March 2022) (except for standard chemicals) were used.

References

1. Ara, T., Enomoto, M., Arita, M., Ikeda, C., Kera, K., Yamada, M., Nishioka, T., Ikeda, T., Nihei, Y., Shibata, D. *et al.* (2015) Metabolonote: a wiki-based database for managing hierarchical metadata of metabolome analyses. *Front. Bioeng. Biotechnol.*, **3**, 38.
2. Kessner, D., Chambers, M., Burke, R., Agus, D. and Mallick, P. (2008) ProteoWizard: open source software for rapid proteomics tools development. *Bioinformatics*, **24**, 2534-2536.
3. Sakurai, N. and Shibata, D. (2017) Tools and databases for an integrated metabolite annotation environment for liquid chromatography-mass spectrometry-based untargeted metabolomics. *Carot. Sci.*, **22**, 16-22.
4. Sakurai, N., Ara, T., Enomoto, M., Motegi, T., Morishita, Y., Kurabayashi, A., Iijima, Y., Ogata, Y., Nakajima, D., Suzuki, H. *et al.* (2014) Tools and databases of the KOMICS web portal for preprocessing, mining, and dissemination of metabolomics data. *BioMed Res. Int.*, **2014**, 194812.
5. Sakurai, N., Narise, T., Sim, J.S., Lee, C.M., Ikeda, C., Akimoto, N., Kanaya, S. and Stegle, O. (2018) UC2 search: using unique connectivity of uncharged compounds for metabolite annotation by database searching in mass spectrometry-based metabolomics. *Bioinformatics*, **34**, 698-700.
6. Kanehisa, M., Sato, Y., Kawashima, M., Furumichi, M. and Tanabe, M. (2016) KEGG as a reference resource for gene and protein annotation. *Nucleic Acids Res.*, **44**, D457-462.
7. Afendi, F.M., Okada, T., Yamazaki, M., Hirai-Morita, A., Nakamura, Y., Nakamura, K., Ikeda, S., Takahashi, H., Altaf-Ul-Amin, M., Darusman, L.K. *et al.* (2012) KNApSAcK family databases: integrated metabolite-plant species databases for multifaceted plant research. *Plant Cell Physiol.*, **53**, e1.
8. Wishart, D.S., Jewison, T., Guo, A.C., Wilson, M., Knox, C., Liu, Y., Djoumbou, Y., Mandal, R., Aziat, F., Dong, E. *et al.* (2013) HMDB 3.0--The Human Metabolome Database in 2013. *Nucleic Acids Res.*, **41**, D801-807.
9. Fahy, E., Subramaniam, S., Murphy, R.C., Nishijima, M., Raetz, C.R., Shimizu, T., Spener, F., van Meer, G., Wakelam, M.J. and Dennis, E.A. (2009) Update of the LIPID MAPS comprehensive classification system for lipids. *J. Lipid Res.*, **50 Suppl**, S9-14.
10. Akimoto, N., Ara, T., Nakajima, D., Suda, K., Ikeda, C., Takahashi, S., Muneto, R., Yamada, M., Suzuki, H., Shibata, D. *et al.* (2017) FlavonoidSearch: A system for comprehensive flavonoid annotation by mass spectrometry. *Sci. Rep.*, **7**, 1243.
11. Sansone, S.A., Rocca-Serra, P., Field, D., Maguire, E., Taylor, C., Hofmann, O., Fang, H.,

Neumann, S., Tong, W., Amaral-Zettler, L. *et al.* (2012) Toward interoperable bioscience data. *Nat. Genet.*, **44**, 121-126.

12. Wang, M., Jarmusch, A.K., Vargas, F., Aksenov, A.A., Gauglitz, J.M., Weldon, K., Petras, D., da Silva, R., Quinn, R., Melnik, A.V. *et al.* (2020) Mass spectrometry searches using MASST. *Nat. Biotechnol.*, **38**, 23-26.
13. Tsugawa, H., Kind, T., Nakabayashi, R., Yukihira, D., Tanaka, W., Cajka, T., Saito, K., Fiehn, O. and Arita, M. (2016) Hydrogen Rearrangement Rules: Computational MS/MS Fragmentation and Structure Elucidation Using MS-FINDER Software. *Anal. Chem.*, **88**, 7946-7958.
14. Lai, Z., Tsugawa, H., Wohlgemuth, G., Mehta, S., Mueller, M., Zheng, Y., Ogiwara, A., Meissen, J., Showalter, M., Takeuchi, K. *et al.* (2018) Identifying metabolites by integrating metabolome databases with mass spectrometry cheminformatics. *Nat. Methods*, **15**, 53-56.
15. Sumner, L., Amberg, A., Barrett, D., Beale, M., Beger, R., Daykin, C., Fan, T.M., Fiehn, O., Goodacre, R., Griffin, J. *et al.* (2007) Proposed minimum reporting standards for chemical analysis. *Metabolomics*, **3**, 211-221.
16. Blaženović, I., Kind, T., Ji, J. and Fiehn, O. (2018) Software Tools and Approaches for Compound Identification of LC-MS/MS Data in Metabolomics. *Metabolites*, **8**, 31.
17. Hiraga, Y., Ara, T., Sato, N., Akimoto, N., Sugiyama, K., Suzuki, H. and Kera, K. (2021) Metabolic analysis of unripe papaya (*Carica papaya* L.) to promote its utilization as a functional food. *Biosci. Biotechnol. Biochem.*, **85**, 1194-1204.
18. Sakurai, N., Mardani-Korrani, H., Nakayasu, M., Matsuda, K., Ochiai, K., Kobayashi, M., Tahara, Y., Onodera, T., Aoki, Y., Motobayashi, T. *et al.* (2020) Metabolome Analysis Identified Okaramines in the Soybean Rhizosphere as a Legacy of Hairy Vetch. *Front. Genet.*, **11**, 114.
19. Horai, H., Arita, M., Kanaya, S., Nihei, Y., Ikeda, T., Suwa, K., Ojima, Y., Tanaka, K., Tanaka, S., Aoshima, K. *et al.* (2010) MassBank: a public repository for sharing mass spectral data for life sciences. *Journal of mass spectrometry : JMS*, **45**, 703-714.
20. Delaporte, G., Cladière, M. and Camel, V. (2019) Untargeted food chemical safety assessment: A proof-of-concept on two analytical platforms and contamination scenarios of tea. *Food Control*, **98**, 510-519.
21. Haug, K., Cochrane, K., Nainala, V.C., Williams, M., Chang, J., Jayaseelan, K.V. and O'Donovan, C. (2020) MetaboLights: a resource evolving in response to the needs of its scientific community. *Nucleic Acids Res.*, **48**, D440-D444.
